# Lentiviral haemopoietic stem/progenitor cell gene therapy for treatment of Wiskott-Aldrich syndrome: interim results of a non-randomised, open-label, phase 1/2 clinical study

**DOI:** 10.1016/S2352-3026(19)30021-3

**Published:** 2019-04-10

**Authors:** Francesca Ferrua, Maria Pia Cicalese, Stefania Galimberti, Stefania Giannelli, Francesca Dionisio, Federica Barzaghi, Maddalena Migliavacca, Maria Ester Bernardo, Valeria Calbi, Andrea Angelo Assanelli, Marcella Facchini, Claudia Fossati, Elena Albertazzi, Samantha Scaramuzza, Immacolata Brigida, Serena Scala, Luca Basso-Ricci, Roberta Pajno, Miriam Casiraghi, Daniele Canarutto, Federica Andrea Salerio, Michael H Albert, Antonella Bartoli, Hermann M Wolf, Rossana Fiori, Paolo Silvani, Salvatore Gattillo, Anna Villa, Luca Biasco, Christopher Dott, Emily J Culme-Seymour, Koenraad van Rossem, Gillian Atkinson, Maria Grazia Valsecchi, Maria Grazia Roncarolo, Fabio Ciceri, Luigi Naldini, Alessandro Aiuti

**Affiliations:** a San Raffaele Telethon Institute for Gene Therapy (SR-Tiget), IRCCS San Raffaele Scientific Institute, Milan, Italy; b Pediatric Immunohematology and Bone Marrow Transplantation Unit, IRCCS San Raffaele Scientific Institute, Milan, Italy; c Hematology and Bone Marrow Transplantation Unit, IRCCS San Raffaele Scientific Institute, Milan, Italy; d Department of Anesthesia and Critical Care, IRCCS San Raffaele Scientific Institute, Milan, Italy; e Blood Transfusion Service, IRCCS San Raffaele Scientific Institute, Milan, Italy; f Vita-Salute San Raffaele University, Milan, Italy; g Center of Biostatistics for Clinical Epidemiology, University of Milano—Bicocca, Monza, Italy; h Department of Pediatric Hematology/Oncology, Dr von Haunersches University Children's Hospital, Munich, Germany; i Clinical Pharmacokinetics Unit, San Matteo Hospital, Pavia, Italy; j Immunology Outpatient Clinic, and Sigmund Freud Private University—Medical School, Vienna, Austria; k Milan Unit, Istituto di Ricerca Genetica e Biomedica, Consiglio Nazionale delle Ricerche, Milan, Italy; l CSD Pharma Consulting, Redhill, UK; m Rare Diseases Unit, GlaxoSmithKline, Brentford, UK; n Division of Stem Cell Transplantation and Regenerative Medicine and Institute for Stem Cell Biology and Regenerative Medicine, Stanford University, Stanford, CA, USA; o University College London, Great Ormond Street Institute of Child Health, Faculty of Population Health Sciences, London, UK; p Orchard Therapeutics, London, UK; q Sangamo Therapeutics, London, UK

## Abstract

**Background:**

Wiskott-Aldrich syndrome is a rare, life-threatening, X-linked primary immunodeficiency characterised by microthrombocytopenia, infections, eczema, autoimmunity, and malignant disease. Lentiviral vector-mediated haemopoietic stem/progenitor cell (HSPC) gene therapy is a potentially curative treatment that represents an alternative to allogeneic HSPC transplantation. Here, we report safety and efficacy data from an interim analysis of patients with severe Wiskott-Aldrich syndrome who received lentiviral vector-derived gene therapy.

**Methods:**

We did a non-randomised, open-label, phase 1/2 clinical study in paediatric patients with severe Wiskott-Aldrich syndrome, defined by either *WAS* gene mutation or absent Wiskott-Aldrich syndrome protein (WASP) expression or a Zhu clinical score of 3 or higher. We included patients who had no HLA-identical sibling donor available or, for children younger than 5 years of age, no suitable 10/10 matched unrelated donor or 6/6 unrelated cord blood donor. After treatment with rituximab and a reduced-intensity conditioning regimen of busulfan and fludarabine, patients received one intravenous infusion of autologous CD34+ cells genetically modified with a lentiviral vector encoding for human *WAS* cDNA. The primary safety endpoints were safety of the conditioning regimen and safety of lentiviral gene transfer into HSPCs. The primary efficacy endpoints were overall survival, sustained engraftment of genetically corrected HSPCs, expression of vector-derived WASP, improved T-cell function, antigen-specific responses to vaccinations, and improved platelet count and mean platelet volume normalisation. This interim analysis was done when the first six patients treated had completed at least 3 years of follow-up. The planned analyses are presented for the intention-to-treat population. This trial is registered with ClinicalTrials.gov (number NCT01515462) and EudraCT (number 2009-017346-32).

**Findings:**

Between April 20, 2010, and Feb 26, 2015, nine patients (all male) were enrolled of whom one was excluded after screening; the age range of the eight treated children was 1·1–12·4 years. At the time of the interim analysis (data cutoff April 29, 2016), median follow-up was 3·6 years (range 0·5–5·6). Overall survival was 100%. Engraftment of genetically corrected HSPCs was successful and sustained in all patients. The fraction of WASP-positive lymphocytes increased from a median of 3·9% (range 1·8–35·6) before gene therapy to 66·7% (55·7–98·6) at 12 months after gene therapy, whereas WASP-positive platelets increased from 19·1% (range 4·1–31·0) to 76·6% (53·1–98·4). Improvement of immune function was shown by normalisation of in-vitro T-cell function and successful discontinuation of immunoglobulin supplementation in seven patients with follow-up longer than 1 year, followed by positive antigen-specific response to vaccination. Severe infections fell from 2·38 (95% CI 1·44–3·72) per patient-year of observation (PYO) in the year before gene therapy to 0·31 (0·04–1·11) per PYO in the second year after gene therapy and 0·17 (0·00–0·93) per PYO in the third year after gene therapy. Before gene therapy, platelet counts were lower than 20 × 10^9^ per L in seven of eight patients. At the last follow-up visit, the platelet count had increased to 20–50 × 10^9^ per L in one patient, 50–100 × 10^9^ per L in five patients, and more than 100 × 10^9^ per L in two patients, which resulted in independence from platelet transfusions and absence of severe bleeding events. 27 serious adverse events in six patients occurred after gene therapy, 23 (85%) of which were infectious (pyrexia [five events in three patients], device-related infections, including one case of sepsis [four events in three patients], and gastroenteritis, including one case due to rotavirus [three events in two patients]); these occurred mainly in the first 6 months of follow-up. No adverse reactions to the investigational drug product and no abnormal clonal proliferation or leukaemia were reported after gene therapy.

**Interpretation:**

Data from this study show that gene therapy provides a valuable treatment option for patients with severe Wiskott-Aldrich syndrome, particularly for those who do not have a suitable HSPC donor available.

**Funding:**

Italian Telethon Foundation, GlaxoSmithKline, and Orchard Therapeutics.

## Introduction

Wiskott-Aldrich syndrome is a rare, X-linked, primary immunodeficiency characterised by microthrombocytopenia, recurrent infections, eczema, and increased risk for autoimmunity and lymphoid malignant diseases.^1^, ^2^ The disease is due to mutations in the *WAS* gene, which encodes the Wiskott-Aldrich syndrome protein (referred to as WASP)—an intracellular key regulator of actin polymerisation.^2^, ^3^ WASP-deficient immune cells have compromised immunological synapsis formation, cell migration, and cytotoxicity.^1^ Survival of patients with Wiskott-Aldrich syndrome is dependent on the severity of the disease. Patients with classic severe phenotype (Zhu clinical score ≥3) have an approximate survival of 15 years with supportive treatment only.^4^, ^5^

Haemopoietic stem/progenitor cell (HSPC) transplantation from an HLA-identical sibling donor is the treatment of choice for patients with Wiskott-Aldrich syndrome, but such a donor is not always available.^6^, ^7^, ^8^, ^9^, ^10^ HSPC transplantation from an HLA-matched unrelated donor can also be curative but can be hampered by development of graft-versus-host disease, graft rejection, or autoimmune complications if complete chimerism is not achieved.^8^ The best outcome for unrelated HSPC transplantation occurs when the recipient is younger than 5 years of age at the time of transplant.^9^, ^10^

An alternative potentially curative option for patients with Wiskott-Aldrich syndrome is gene therapy, consisting of a reduced intensity conditioning regimen followed by infusion of ex-vivo genetically corrected autologous HSPCs. Ex-vivo gene therapy has several potential advantages, including absence of graft-versus-host disease and decreased toxicity due to the reduced intensity conditioning regimen. In a previous trial using a γ-retroviral vector under the control of a strong viral promoter, gene therapy was feasible and resulted in functional correction of blood cell defects.^11^ However, insertions of the γ-retroviral vector were clustered in proto-oncogenes; integration-driven overexpression of these genes triggered the development of severe side-effects such as leukaemia and myelodysplasia in most patients.^11^

**Research in context**

**Evidence before this study**

We searched PubMed from database inception to Nov 25, 2018, for relevant studies of treatments for Wiskott-Aldrich syndrome, including acronyms, synonyms, and closely related words for the terms “Wiskott-Aldrich syndrome”, “gene therapy”, and “haematopoietic stem cell transplantation”. We did not restrict our search by study design or language. We identified further studies by searching relevant websites and the reference lists of reports identified by our search. The only curative treatment for Wiskott-Aldrich syndrome is allogeneic haemopoietic stem/progenitor cell (HSPC) transplantation with a suitable HLA-matched donor. However, many patients do not have such a donor available, and treatment can be hampered by development of graft-versus-host disease, graft rejection, and autoimmune complications if complete chimerism is not achieved. Proof-of-principle for an autologous approach with gene therapy came from a previous trial using a γ-retroviral vector carrying a functional *WAS* gene under the control of a strong viral promoter. However, integration-driven overexpression of proto-oncogenes triggered development of severe side-effects such as leukaemia and myelodysplasia in most patients. To address the safety issues with γ-retroviral vectors for Wiskott-Aldrich syndrome, a self-inactivating lentiviral vector was developed, encoding for human Wiskott-Aldrich syndrome protein (WASP) under the control of a 1·6 kb reconstituted *WAS* gene promoter. In this construct the transgene is expressed in the context of the proper endogenous regulatory elements, rather than a viral promoter. A phase 1/2 clinical study on this lentiviral vector-derived gene therapy combined with a reduced-intensity conditioning regimen was initiated in patients with Wiskott-Aldrich syndrome in 2010, and preliminary results for the first three patients treated (follow-up of 20–32 months) were reported in 2013. An additional study, using the same lentiviral vector construct but manufactured by a different process reported treatment of seven patients with Wiskott-Aldrich syndrome, with follow-up of 7–42 months.

**Added value of this study**

We report findings of an interim analysis of the phase 1/2 clinical study that was initiated in 2010. Eight patients with Wiskott-Aldrich syndrome are included in the analysis of safety and efficacy data, with median follow-up of 3·6 years (range 0·5–5·6), the longest follow-up published to date. After a reduced-intensity conditioning regimen and subsequent infusion of transduced CD34+ cells, all eight patients showed good haematological reconstitution and sustained engraftment of transduced cells. WASP expression was increased in peripheral blood myeloid and lymphoid lineages and in platelets. Significant improvement of immune function was seen by normalisation of in-vitro T-cell function, pronounced reduction of severe infections, and successful discontinuation of immunoglobulin supplementation followed by response to vaccination. Platelet counts increased substantially, resulting in platelet transfusion independence and absence of severe bleeding events.

**Implications of all the available evidence**

Lentiviral vector-mediated HSPC gene therapy is an alternative, potentially curative, treatment for patients with Wiskott-Aldrich syndrome that could be administered to a wider range of patients than allogeneic HSPC transplantation and might result in fewer complications. This possibility will be confirmed by longer follow-up of patients treated with gene therapy.

To address the safety issues with γ-retroviral vectors for Wiskott-Aldrich syndrome, we developed a self-inactivating lentiviral vector encoding human WASP under the control of a 1·6 kb reconstituted *WAS* gene promoter.^12^ The use of this endogenous promoter ensures that the transgene is expressed in the context of the proper endogenous regulatory elements.^3^ Its moderate enhancer activity combined with the self-inactivating long-terminal repeat design and absence of preference to integrate in or near oncogenes reduces the risk of insertional mutagenesis, as shown by in-vitro transformation assays^13^ and preclinical in-vivo studies in WASP-deficient mice.^14^, ^15^

In a phase 1/2 clinical study, autologous CD34+ cells genetically modified with a lentiviral vector encoding for human *WAS* cDNA^12^, ^14^ were re-infused to patients with severe Wiskott-Aldrich syndrome after a reduced-intensity conditioning regimen.^16^ Preliminary data from the initial follow-up (20–32 months) of the first three patients in the study have been reported.^16^ Here, we report data from an interim analysis that was planned in the study protocol to be done after the first six patients treated had completed at least 3 years of follow-up. This interim analysis was agreed with the European Medicines Agency (EMA) and its paediatric committee (PDCO) as adequate to support a marketing authorisation application. Further analyses are planned when all patients in the study have completed 3 years of follow-up and again when all patients have completed at least 8 years of follow-up.

## Methods

### Study design and participants

We undertook an open-label, non-randomised, phase 1/2 clinical study at the Pediatric Clinical Research Unit and Pediatric Immunohematology and Bone Marrow Transplantation Unit of the San Raffaele Scientific Institute (Milan, Italy). The drug product (autologous CD34+ cells genetically modified with a lentiviral vector encoding for human *WAS* cDNA), trial design, and procedures have been described previously (appendix pp 1–3).^16^ Patients were eligible for the study if they had Wiskott-Aldrich syndrome defined by a *WAS* genetic mutation, with absent WASP expression, severe *WAS* mutation,^2^ or severe clinical phenotype (Zhu clinical score ≥3).^3^ We included patients if they had no HLA-identical sibling donor or, for children younger than 5 years of age, no suitable 10/10 matched unrelated donor or 6/6 unrelated cord blood donor.^9^ We excluded patients who either had HIV infection, neoplasia, cytogenetic alterations typical of myelodisplastic syndrome or acute myeloid leukaemia, or end-organ functions or any other severe disease which, in the judgment of the investigator, would have been inappropriate for entry into this study. We also excluded patients who had undergone an allogeneic HSPC transplant in the previous 6 months or earlier and had evidence of residual cells of donor origin. The conditions required by the study protocol for enrolment of patients have been assessed and fulfilment of the inclusion and exclusion criteria have been assessed in the screening phase, during which time eligible patients underwent clinical, laboratory, and instrumental pretreatment work-up, including additional disease-specific assessments. We obtained a bone marrow aspirate to assess morphology, cellularity, CD34+ cell content, and clonogenic activity.

The protocol and other trial-related materials were approved by the independent ethics committee of the San Raffaele Scientific Institute and the Italian regulatory authority (Agenzia Italiana del Farmaco [AIFA]). Written informed consent was provided by parents or legal representatives of patients before initiation of study-specific procedures.

For the assessment of WASP expression and T lymphocyte proliferative responses, we obtained peripheral blood samples from healthy controls, in accordance with the Declaration of Helsinki. Informed consent was approved by the institutional ethics committee of the San Raffaele Hospital in 2009 (Tiget Periblood protocol).

### Procedures

We obtained a backup of autologous HSPCs and cryopreserved them approximately 5–10 weeks before the planned day of gene therapy, either by bone marrow harvest or by leukapheresis, after administration of granulocyte colony-stimulating factor (in most cases, lenograstim), with or without plerixafor. If the HSPC source was mobilised peripheral blood, we also gathered cells for future transduction at this time and cells were cryopreserved until the start of gene transduction on day −3. If the HSPC source was bone marrow, we obtained cells from iliac crests under general anaesthesia on day −3.

We administered intravenous CD20 monoclonal antibody (rituximab 375 mg/m^2^) on day −22. The reduced-intensity conditioning regimen comprised eight doses of intravenous busulfan administered every 6 h from days −3 to −1 (plus an optional ninth dose to reach the target cumulative busulfan area under the curve [AUC], if needed) and two doses of intravenous fludarabine (30 mg/m^2^ per day) on days −3 and −2. We monitored busulfan pharmacokinetics and adjusted the dose to avoid excessive toxicity or insufficient exposure.^17^ Initially, the target busulfan AUC range was 4500–6000 ng × h / mL per dose, equivalent to a cumulative target of 36 000–48 000 ng × h / mL. We amended the cumulative busulfan AUC target to 48 000 (±10%) ng × h / mL after treatment of six patients. The actual doses and AUC of busulfan received by each patient are summarised in the appendix (p 13). One intravenous infusion of autologous CD34+ cells obtained from bone marrow harvest or mobilised peripheral blood stem cells and transduced with the *WAS* lentiviral vector^12^, ^14^ was given on day 1. The planned target dose was 5–10 × 10^6^ CD34+ cells per kg, with an acceptable range of 2–20 × 10^6^ cells per kg. CD34+ cell manipulation and transduction procedures were done at MolMed SpA (Milan, Italy), as described previously.^16^ Patients were in hospital for a median of 52 days (range 36–82), during which time they underwent chemotherapy, gene therapy, and short-term follow-up, then they were followed up as outpatients unless invasive procedures were needed or complications arose. Clinical examinations were scheduled during screening, at baseline (timing of baseline varied by patient and was from the end of screening to the day before harvest of peripheral blood cells or the day before rituximab administration [which was on day −22]), day −14, day 1 (when gene therapy was initiated), day 7, day 14, day 21, day 30, day 60, day 90, day 180, and year 1, year 1·5, year 2, year 2·5, year 3, and annually to year 8. After treatment, patients' assessment also included routine laboratory tests, microbiological tests, diagnostic imaging, immune profiling, and specific immunological tests, according to the time schedule defined in the study protocol. All adverse events—regardless of seriousness or relation to the drug product—have been recorded in trial case report forms, and the investigators have kept detailed records of all reported adverse events. We used Common Terminology Criteria for Adverse Events for grading of adverse events, or clinical judgment if this grading was not applicable.

We assessed quality of life with a questionnaire (appendix pp 1, 2) given to patients' parents, which was composed of questions about lifestyle and life in a protected environment (eg, hygiene restrictions, food, contact with other children), attendance at school (according to age), social ability with peers, and practice of sport activities (according to age). The questionnaire was given to parents before treatment and then every year at the follow-up visit (starting in year 1 after gene therapy).

### Outcomes

The primary study objectives were to assess the safety of drug product administration after a reduced-intensity conditioning regimen, the long-term engraftment of WASP-expressing gene-transduced cells, and the efficacy of gene therapy in terms of improvement in immune function and thrombocytopenia. Secondary study objectives were to assess the efficacy of gene therapy in improving the patient's clinical condition with respect to severe infections, bleeding episodes, autoimmunity measures, and eczema.

Details of safety and efficacy endpoints are in the appendix (p 1). The primary safety endpoints were safety of the conditioning regimen and short-term and long-term safety of lentiviral gene transfer into HSPCs. The primary efficacy endpoints were overall survival, sustained engraftment of genetically corrected HSPCs, expression of vector-derived WASP, improved T-cell function, antigen-specific responses to vaccinations, and improved platelet count and mean platelet volume normalisation. The endpoints for this study are the same as those defined after initial follow-up of the first three patients treated, reported previously.^16^

### Statistical analysis

The sample size was calculated based on the low prevalence of Wiskott-Aldrich syndrome and the degree of novelty of the proposed experimental approach. Thus, we planned to treat eight patients over an estimated 5-year recruitment period. Planned analyses are presented for the intention-to-treat population, defined as all patients treated with lentiviral gene therapy in the study, and consider data available within the clinical database. Data are summarised using descriptive statistics. Adverse events, serious adverse events, infections, bleeding events, number of platelet transfusions, days in hospital, and anti-infective treatments are summarised as rates (number of events or treatments per patient-year of observation [PYO]). We calculated 95% CIs for crude rates based on the Poisson exact method. We used Medidata Rave 2017.2.3 software to collect data in this study (via electronic case report forms). For analysis of descriptive statistics we used SAS version 9.4, and for data analysis and graphical representation of data we used Microsoft Excel 365 and Graph Pad Prism version 5 for Mac OS X.

This trial is registered with ClinicalTrials.gov (number NCT01515462) and EudraCT (number 2009-017346-32).

### Role of the funding source

The funder had no role in study design but did contribute to protocol amendments. The funder had a role in data collection, data analysis, data interpretation, and writing of the report. The corresponding author had full access to all data in the study and had final responsibility for the decision to submit for publication.

## Results

**Figure 1:**
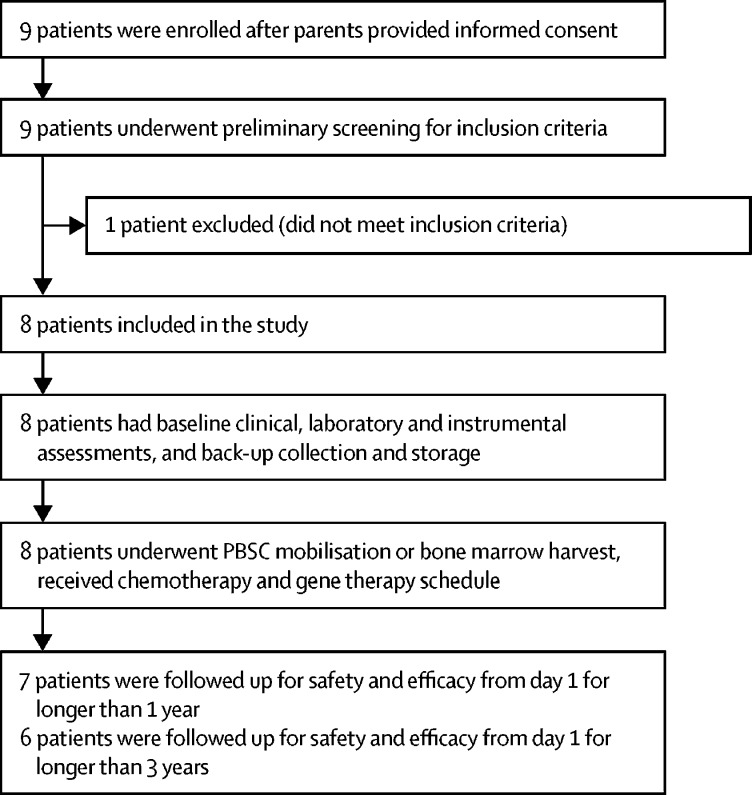
Study design PBSC=peripheral blood stem cell.

The clinical protocol was approved by the Italian regulatory authority (AIFA) on March 15, 2010. Between April 20, 2010, and Feb 26, 2015, nine patients (all male) were enrolled to the study, of whom eight received gene therapy. The fifth patient enrolled was withdrawn during screening because he had a revertant mutation in more than 5% of lymphoid cells, which was an exclusion criterion at that time (figure 1). The protocol was subsequently amended in April, 2014, to allow treatment of subsequent patients with revertant mutations (approved by the San Raffaele Scientific Institute ethics committee on April 14, 2014, and by AIFA on April 17, 2014).

At the time of this planned interim analysis (data cutoff April 29, 2016), median follow-up was 3·6 years (range 0·5–5·6). Six patients had at least 3 years of follow-up; of the other two patients, one had 1·5 years of follow-up and the other had 6 months of follow-up (appendix p 14). All eight patients were alive and well at the time of this interim analysis.

The median age of patients on the day of gene therapy was 2·2 years (range 1·1–12·4). Baseline characteristics for each patient are summarised in table 1. The observed *WAS* gene mutations were nonsense, deletions, missense, and a novel complete inversion of exons 1–7 that included part of the promoter region.^18^ All eight patients had severe clinical disease before gene therapy (Zhu clinical score range 3–5A). Before gene therapy, five patients had less than 5% of peripheral blood lymphocytes expressing WASP and three patients had residual WASP expression (20–36% of peripheral blood lymphocytes). All patients (n=8) had recurrent infections, eczema, thrombocytopenia, bleeding, and immune disorders (eg, allergies and autoimmune or autoinflammatory manifestations; table 1).

**Table 1:** Baseline clinical characteristics of patients with Wiskott-Aldrich syndrome and drug product characteristics

	**Patient 1**	**Patient 2**	**Patient 3**	**Patient 4**	**Patient 6**	**Patient 7**	**Patient 8**	**Patient 9**
Infections	Recurrent ENT infections, bronchiolitis, frequent viral infections (CMV, VZV, HSV, EBV)	Severe infections (pneumonia, colitis, arthritis, cellulitis, CVC-related), chronic CMV infection, URTI, UTI	Pneumonia with respiratory distress (*Pneumonia jiroveci* plus CMV), chronic CMV infection, URTI, otitis media	Neonatal sepsis, candida infection, chronic CMV infection, viral enteritis	Pneumonia, URTI, bacterial conjunctivitis, bacterial UTI, bacterial and viral gastroenteritis, folliculitis, candida infection	Recurrent respiratory-tract infections, otitis media, bacterial gastrointestinal infection, colitis or gastroenteritis	Recurrent respiratory-tract and skin infections, pneumonia, severe eye infections with residual visual impairment	Severe episodes of bacterial and viral enteritis, pneumonia, VZV infection
Bleeding events	Skin petechiae	Skin petechiae, gastrointestinal bleeding, conjunctival bleeding	Skin petechiae, gastrointestinal bleeding, epistaxis	Skin petechiae, gastrointestinal bleeding, epistaxis, signs of CNS microhaemorrhages, CVC-related bleeding	Skin purpura or petechiae, gastrointestinal bleeding, mucosal bleeding, epistaxis, conjunctival bleeding	Skin purpura or petechiae, gastrointestinal bleeding, mucosal bleeding	Skin petechiae, gastrointestinal bleeding, epistaxis, eye bleeding, mucosal bleeding	Gastrointestinal bleeding, haematuria, epistaxis, conjunctival bleeding
Eczema score*	3 (moderate)	2 (mild)	4 (severe)	3 (moderate)	2 (mild)	2 (mild)	2 (mild)	2 (mild)
Other	Developmental disorder, allergy	Failure to thrive, elevated inflammatory indexes or vasculitis, hepatosplenomegaly, allergy	Gastro-oesophageal reflux or food aversion (fed through a nasogastric tube), food or drug allergy, mild developmental delay	Severe refractory autoimmune thrombocytopenia, food or drug allergy with anaphylaxis	Food allergy, hepatomegaly, splenomegaly, inflammatory lymphadenopathy, eosinophilia	Suspected food allergy	Food allergy	Recurrent arthritis and vasculitis, Henoch-Schonlein purpura with nephritic-nephrotic syndrome, panuveitis with visual impairment, severe Crohn-like enterocolitis, perianal fistulae and abscesses, pyoderma gangrenosum
*WAS* gene mutation (rs number)	Exon10, 995C→T (Arg321X) in cDNA(rs782802310)†	1337–1338 + 9del in cDNA(rs number awaited)	Exon1, 37C→T (Arg13X) in cDNA(rs 193922415)	Exon1, 91G→A in cDNA(rs782730988)	Exon 10, 1595del, proximal breakpoint (5247_6842del) in genomic DNA(rs number awaited)	Exon12, 1509A→T in cDNA(rs1289921805)	735-2A→G in cDNA(rs number awaited)	inv(X)(5721;11840)^18^ in genomic DNA (rs number awaited)
Type of mutation	Nonsense	Deletion	Nonsense	Missense	Deletion	Nonstop or readthrough	Splice site	Inversion
Peripheral blood lymphocytes expressing WASP (%)‡	<5%	<5%	<5%	<5%	35·6%§	<5%	20·8% (revertant cell population)	3·4%
Zhu clinical score	3	4	4	5	4	3	4	5A
Age on day of gene therapy (years)	5·9	1·6	1·1	2·4	1·9	1·9	11·1	12·4
Source of transduced CD34+ cells	Bone marrow, mobilised peripheral blood¶‖	Bone marrow	Bone marrow	Bone marrow	Bone marrow	Bone marrow	Mobilised peripheral blood¶	Mobilised peripheral blood**
Total cell dose CD34+ (×10^6^ per kg)	3·7, 5·3‖	14·1	10·2	10·3	7·8	7·8	7·0	16·8
Transduction efficiency (%)	92%, 88%‖	97%	100%	94%	93%	91%	96%	88%
Vector copy number per genome	1·9, 1·4‖	2·4	2·8	2·3	2·3	4·3	3·2	3·0

Patients 1, 2, 3, and 9 have been described previously.^16^, ^18^ Patient 5 was enrolled but subsequently withdrawn during screening. ENT=ear, nose, and throat. CMV=cytomegalovirus. VZV=varicella zoster virus. HSV=herpes simplex virus. EBV=Epstein-Barr virus. CVC=central venous cathether. URTI=upper respiratory-tract infection. UTI=urinary-tract infection. WASP=Wiskott-Aldrich syndrome protein.

* Eczema scores were 1 (absent), 2 (mild), 3 (moderate), and 4 (severe); see appendix pp 1–3.

† Patient 1 also had a variant of unknown significance (non-pathogenic mutation in exon 6, 572C→A [His180Asn]).

‡ Measured by fluorescence-activated cell sorting.

§ Undetectable by Western blot.

¶ Obtained after administration of lenograstim.

‖ Patient 1 received both bone marrow and mobilised peripheral blood; transduction was done separately, and drug product characteristics are reported for both.

** Obtained after administration of lenograstim and plerixafor.

The CD34+ cell source was bone marrow for five patients, mobilised peripheral blood for two patients, and a combination of both for one patient. Mobilised peripheral blood stem cells were obtained after administration of either lenograstim (n=2) or lenograstim and plerixafor (n=1). The dose of drug product administered was 7·0–16·8 × 10^6^ CD34+ cells per kg. CD34+ cells sourced from bone marrow had a transduction efficiency of 91–100%, with an average vector copy number per genome of 1·9–4·3. CD34+ cells sourced from peripheral blood had a transduction efficiency of 88–96% with an average vector copy number per genome of 1·4–3·2 (table 1).

The reduced-intensity conditioning regimen was not associated with any unexpected toxic effects during the first 100 days after gene therapy. All eight patients had severe neutropenia, with an absolute neutrophil count of 0·2 × 10^9^ cells per L or lower (appendix p 13). All eight patients had adverse events during follow-up but none of the events was judged by the investigator to be related to the drug product. No patient had severe mucositis.

Engraftment was successful in all eight patients (absolute neutrophil count ≥0·5 × 10^9^ cells per L within 60 days; appendix pp 4, 13) without the need for backup autologous cell administration. Time to neutrophil engraftment ranged from 18 days to 59 days. One patient (patient 4) needed administration of granulocyte colony-stimulating factor (on day 42 and day 44) for delayed neutrophil recovery.

The incidence of adverse events and serious adverse events was highest in the first 6 months of follow-up then declined and reached a plateau from 6 months after gene therapy onwards (table 2). The rate of serious adverse events per PYO declined from 4·8 (95% CI 2·9–7·4) during the first 6 months of follow-up after gene therapy to 0·6 (0·2–1·6) between 1 year and 2 years of follow-up and to 0·2 (0·0–0·9) between 2 years and 3 years of follow-up. Likewise, the rate of adverse events per PYO declined from 65·5 (95% CI 57·8–73·9) during the first 6 months of follow-up after gene therapy to 22·1 (17·5–27·6) between 6 months and 12 months of follow-up and to 15·8 (12·8–19·3) between 2 years and 3 years of follow-up. No adverse event or serious adverse event was considered to be related to drug product by the investigator. Rates of adverse events commonly associated with Wiskott-Aldrich syndrome (eg, bleeding events, eczema, and infections) declined after gene therapy. 27 serious adverse events in six patients were reported after gene therapy, of which 23 (85%) were of infectious origin, including pyrexia (five events in three patients), device-related infections including one case of sepsis (four events in three patients), and gastroenteritis of which one case was due to rotavirus (three events in two patients; table 2).

**Table 2:** Serious adverse events after gene therapy

		**0–6 months after gene therapy (n=8)**		**6–12 months after gene therapy (n=8)**		**1–2 years after gene therapy (n=7)**		**2–3 years after gene therapy (n=6)**		≥3 years after gene therapy (n=5)*		**Total after gene therapy (n=8)**	
		Patients	Events	Patients	Events	Patients	Events	Patients	Events	Patients	Events	Patients	Events
Any serious adverse event		6	19	1	1	3	4	1	1	1	2	6	27
Infection-related event													
	Pyrexia	2	2	1	1	1	2	0	0	0	0	3	5
	Device-related infection	2	2	0	0	1	1	0	0	0	0	2	3
	Acute respiratory distress syndrome†	1	1	0	0	0	0	0	0	0	0	1	1
	Aspergillus infection	1	1	0	0	0	0	0	0	0	0	1	1
	Bacterial sepsis†	1	1	0	0	0	0	0	0	0	0	1	1
	Device-related sepsis	1	1	0	0	0	0	0	0	0	0	1	1
	Disseminated intravascular coagulation†	1	1	0	0	0	0	0	0	0	0	1	1
	Influenza	1	1	0	0	0	0	0	0	0	0	1	1
	Interstitial lung disease	1	1	0	0	0	0	0	0	0	0	1	1
	Gastroenteritis	1	2	0	0	0	0	0	0	0	0	1	2
	Pneumonia aspiration	1	1	0	0	0	0	0	0	0	0	1	1
	Viral infection	1	1	0	0	0	0	0	0	0	0	1	1
	Gastroenteritis rotavirus	0	0	0	0	1	1	0	0	0	0	1	1
	Tooth abscess	0	0	0	0	0	0	1	1	0	0	1	1
	Cellulitis	0	0	0	0	0	0	0	0	1	1	1	1
	Pneumonia bacterial	0	0	0	0	0	0	0	0	1	1	1	1
Other type of event													
	Electrolyte imbalance	1	1	0	0	0	0	0	0	0	0	1	1
	Food allergy	1	1	0	0	0	0	0	0	0	0	1	1
	Irritability	1	1	0	0	0	0	0	0	0	0	1	1
	Neutropenia	1	1	0	0	0	0	0	0	0	0	1	1

MedDRA (version 19.0) preferred terms are used. MedDRA=Medical Dictionary for Regulatory Activities.

* Range 3·0–5·6 years.

† These three events were linked, occurring in patient 2, and were considered to be part of the same condition.

Transient decreases in haematological indices were noted after the conditioning phase in all eight patients. Results of serum chemistry tests did not show any consistent patterns of clinical concern (data not shown).No adverse reactions to the drug product were reported within 48 h of infusion. No adverse findings were reported from replication competent lentivirus assessments and no evidence was seen of abnormal clonal proliferation or leukaemia development after gene therapy. No antibody against WASP was detected in any patient after gene therapy.

Data for the first six patients with at least 3 years of follow-up were analysed, and 184 745 unique insertion sites were identified from bone marrow CD34+ cells, bone marrow precursors, and peripheral blood mature cells from lymphoid and myeloid lineages at different timepoints after gene therapy. The diversity of the clonal repertoire of engineered cells was maintained at a high complexity level starting from around 1 year after gene therapy without major inbalances up to and including the latest follow-up analysed (appendix p 5). The common insertion sites detected in all six patients reflect the classic insertional pattern of lentiviral vectors, including *PACS1* and *KDM2A* previously reported in metachromatic leukodystrophy,^19^ adrenoleukodystrophy, and β thalassemia^20^ gene therapy trials and not associated with clonal expansion. No enrichment for genomic areas associated with insertional mutagenesis events was detected when comparing data at 12–18 months and 30–36 months after gene therapy (appendix p 6).

**Figure 2:**
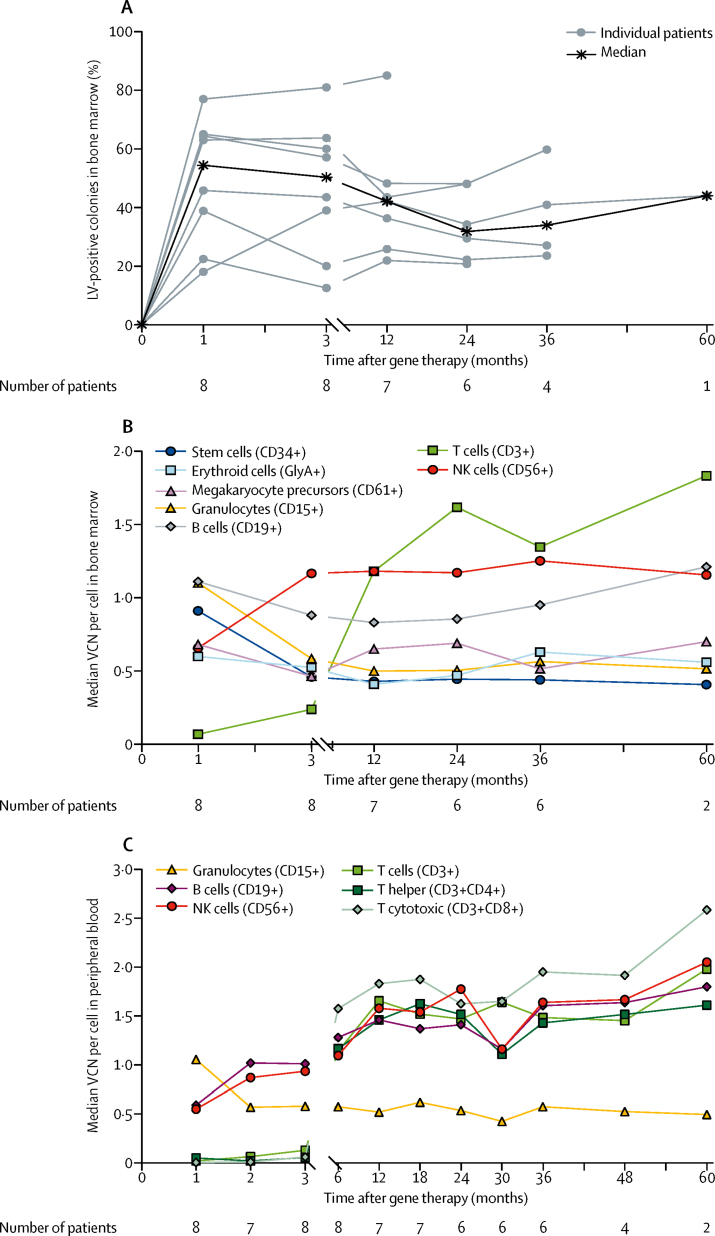
Multilineage engraftment of genetically corrected haemopoietic stem cells and peripheral blood cells in eight patients with Wiskott-Aldrich syndrome treated with lentiviral vector gene therapy (A) In-vivo engraftment of transduced progenitor cells in bone marrow (LV-positive colonies). (B) Median VCN per cell in bone marrow cell lineages. (C) Median VCN per cell in peripheral blood cell lineages. No vector was detected in bone marrow total cells analysed before gene therapy. LV=lentiviral vector. VCN=vector copy number. GlyA=glycophorin A. NK=natural killer.

All seven patients who were followed up for at least 1 year after gene therapy (figure 1) had adequate engraftment of genetically corrected colony-forming cells in bone marrow (figure 2A). Engraftment was sustained over time in both bone marrow and peripheral blood cell lineages (figures 2B, 2C). Gene-corrected granulocytes (CD15+), megakaryocytic precursors (CD61+), erythroid cells (glycophorin A-positive [GlyA+]), B cells (CD19+), and natural killer (NK) cells (CD56+) were detectable from 1 month after gene therapy. T-cell engraftment was slower compared with other lineages, as expected by the required time for T-cell development through the thymus, with transduced T cells appearing from 3 months after gene therapy. Transduced cell engraftment was higher in lymphoid lineages (CD3+, CD4+, CD8+, CD19+, and CD56+) compared with myeloid lineages (CD15+, CD61+, and GlyA+) because of their known selective advantage.

**Figure 3:**
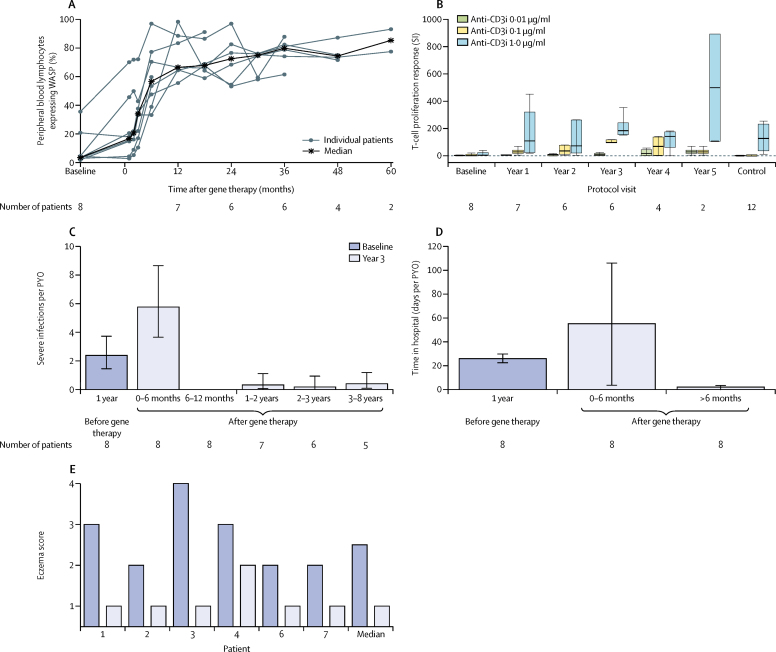
Immune reconstitution and clinical benefit Gene therapy was administered on day 1 (month 0). (A) Percentage of peripheral blood lymphocytes expressing WASP, as measured by flow cytometry. (B) T-cell proliferation response (expressed as SI) after incubation with increasing concentrations of anti-CD3i antigen, by observation period. Boxes represent upper and lower quartiles (outliers excluded). Horizontal line within the box is the median. Whiskers represent most extreme points ≤1·5 × IQR. Control data are from 12 healthy children. (C) Rate of severe infections (events per PYO) for each observation period. Error bars represent the 95% CI. (D) Number of days in hospital (days per PYO) for each observation period. Error bars represent the 95% CI. (E) Eczema scores are 1 (absent), 2 (mild), 3 (moderate), and 4 (severe). Six patients were followed up for at least 3 years after gene therapy. WASP=Wiskott-Aldrich syndrome protein. SI=stimulation index. Anti-CD3i=anti-immobilised CD3. PYO=patient-year of observation.

Lymphocyte counts decreased after conditioning, as expected, then increased progressively. At the latest follow-up visit, T-cell counts (both CD4+ helper and CD8+ cytotoxic T lymphocytes), B-cell counts, and NK cell counts were normal for age in all eight patients,^21^ apart from one patient's (patient 7) total and CD8+ T-cell counts, which were just below the normal range (appendix pp 15–20). Naive CD4+ T cells were normal in four of eight patients at the last follow-up visit, similar to pretreatment levels (appendix p 21).^22^ The fraction of lymphocytes expressing WASP was substantially improved in all eight patients, with a ten times increase in the median value compared with baseline seen at 3 months after gene therapy (range 11–72%) and a 20 times increase recorded consistently at 12 months after gene therapy (figure 3A). The fraction of WASP-positive lymphocytes increased from a median of 3·9% (range 1·8–35·6) before gene therapy to 66·7% (55·7–98·6) at 12 months after gene therapy. A selective advantage was noted in lymphoid lineages, particularly T cells, which showed a higher level of expression compared with myeloid lineages (appendix pp 7, 8). The T-cell proliferative response to anti-immobilised CD3 (anti-CD3i) was severely compromised in all eight patients before gene therapy and steadily improved after gene therapy. In the six patients with 3 years of follow-up, the proliferative response at 3 years was comparable with the response in T cells from healthy controls (figure 3B). The proliferative response to phytohaemagglutinin was normal in seven of the eight patients and returned to normal levels in all seven patients with follow-up at least 1 year after gene therapy, including the patient with abnormal levels before gene therapy.

The improvement in immune function was accompanied by clinical benefit. The rate of severe infections increased in the first 6 months after gene therapy, in accordance with the effects of the conditioning regimen, but declined strikingly thereafter to approximately an order of magnitude lower than the rate in the year before treatment (figure 3C). Severe infections fell from 2·38 (95% CI 1·44–3·72) events per PYO in the year before gene therapy to 0·31 (0·04–1·11) events per PYO in the second year after gene therapy and 0·17 (0·00–0·93) events per PYO in the third year after gene therapy. No reactivation of cytomegalovirus was detected after 6 months of follow-up in three patients who had chronic cytomegalovirus infection before treatment. The rate of anti-infective drug use 2–3 years after gene therapy was approximately four times lower than the rate in the year before treatment (11·3 treatments per PYO *vs* 42·0 treatments per PYO). The number of days spent in hospital also declined substantially from 6 months after gene therapy onwards (figure 3D). Eczema scores improved in all six patients who reached at least 3 years of follow-up, and eczema had completely resolved in five patients by 3 years (figure 3E).

At the time of this interim analysis, intravenous immunoglobulin had been discontinued in seven patients with follow-up longer than 1 year (median time to discontinuation after gene therapy, 1·7 years [IQR 0·9–3·0]). Serum immunoglobulin levels before and after gene therapy are reported in the appendix (p 22). Immunological status was considered to be sufficiently improved to allow the start of childhood vaccinations in five patients. A protective antibody response was shown in all five patients to tetanus toxoid, diphtheria, *Haemophilus influenzae* type B, and hepatitis B. Four of five patients also had a protective response to *Bordetella pertussis*. Protective levels of IgG antibodies against *Streptococcus pneumoniae* were detected in one of five assessable patients after polysaccharide pneumococcal vaccine and in three of four assessable patients after pneumococcal conjugated vaccine (appendix pp 23, 24).

**Figure 4:**
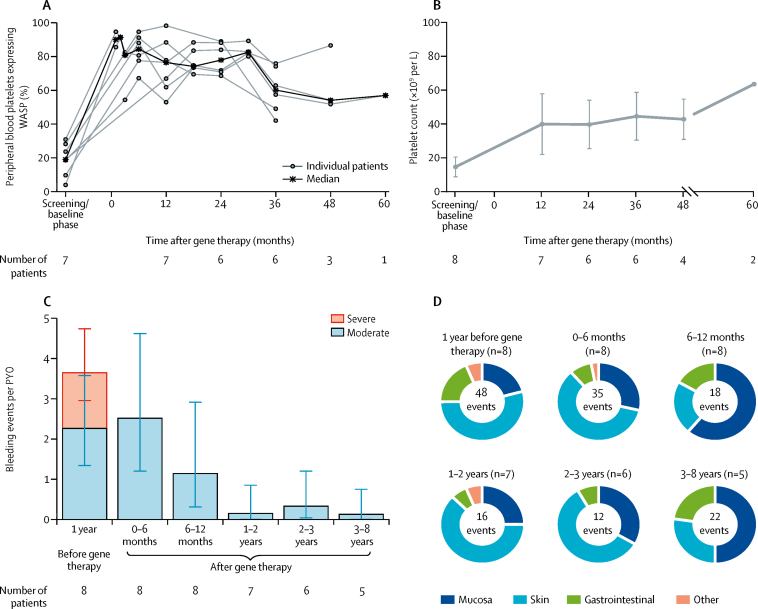
Platelets and bleeding events Gene therapy was administered on day 1 (month 0). (A) Percentage of platelets expressing WASP, as measured by flow cytometry. (B) Mean (SD) platelet count (× 109 cells per L). SD is not plotted for follow-up at 60 months because data were available for only two patients. (C) Moderate or severe bleeding events (per PYO) for each observation period. Error bars represent the 95% CI. (D) Distribution of all bleeding events for each observation period, by body site. Total number of events for each observation period is shown in the centre of each chart. WASP=Wiskott-Aldrich syndrome protein. PYO=patient-year of observation.

The proportion of platelets expressing WASP, as measured by flow cytometry, progressively improved over time in all eight patients after gene therapy (figure 4A; appendix pp 9, 10). WASP-positive platelets increased from 19·1% (range 4·1–31·0) before gene therapy to 76·6% (53·1–98·4) at 12 months after gene therapy. The ratio of mean fluorescence intensity in platelets of patients normalised to those of healthy controls increased from a median of 0·18 (0·01–0·31) before gene therapy to 0·43 (0·20–1·03) at 1 year after gene therapy. A substantial improvement in platelet count was also seen (figure 4B). Before gene therapy, platelet counts were lower than 20 × 10^9^ per L in seven of eight patients. At the last follow-up visit, the platelet count had increased to 20–50 × 10^9^ per L in one patient, 50–100 × 10^9^ per L in five patients, and more than 100 × 10^9^ per L in two patients, which resulted in independence from platelet transfusions and absence of severe bleeding events, although platelets counts remained below the lower limit of the normal range in most patients. Mean platelet volume, which is characteristically reduced in patients with Wiskott-Aldrich syndrome, was within the normal range (7·4–10·9 fL) in seven assessable patients at 1 year after gene therapy and remained at this level except for a transient dip for one patient (patient 6) at year 2. All eight patients needed platelet transfusions before gene therapy (2·5 transfusions per PYO in the year before enrolment), falling to less than 0·3 transfusions per PYO for observation periods beyond 1 year, when transfusions were given only as a precautionary measure for surgery or trauma. The transfusion rate was higher in the preparatory period before gene therapy (42·9 transfusions per PYO, equal to 3·6 transfusions per person-month of observation), mainly because of standard use as bleeding prophylaxis for invasive procedures such as bone marrow aspiration or harvest, central venous cathether positioning, and peripheral blood stem cell leukoapheresis. Two patients (patients 8 and 9) who received mobilised peripheral blood-derived drug product had a faster increase in platelet count after gene therapy compared with those who received bone marrow-derived drug product (appendix pp 9, 10). None of the treated patients required splenectomy.

The improvement in platelet count was accompanied by a reduction in the rate and severity of bleeding events (figure 4C). No severe bleeding events were reported after gene therapy, and a pronounced reduction in moderate bleeding events was also seen after gene therapy. Bleeding events after gene therapy were predominantly related to the skin or mucosa and none resulted in hospitalisation (figure 4D).

Patient 4 had severe refractory autoimmune thrombocytopenia^23^ before gene therapy and was treated both before and after gene therapy with immunosuppressive drugs (high-dose intravenous immunoglobulin, steroids, and rituximab) and with thrombopoietin receptor agonists for 8 months after gene therapy. This patient became independent of platelet transfusions after gene therapy, with only one transfusion in the 1–2 year observation period and one transfusion in the 4–5 year observation period; these transfusions were precautionary because of a surgical procedure and head trauma, respectively. Patient 2 developed severe autoimmune thrombocytopenia approximately 3 months after gene therapy, which resolved within 6 months. Patient 9 had a prolonged history of inflammatory and autoimmune manifestations (Crohn-like enterocolitis, pyoderma gangrenosum, and arthritis) requiring multiple chronic treatments before gene therapy.^18^ However, disease symptoms and signs fully resolved after gene therapy (appendix p 11). A panel of 15 serum autoantibodies were tested before and after gene therapy. The incidence of autoimmune antibodies was highest at baseline (ten positive reports in four of eight patients) but declined by year 1 (two positive reports in two of eight patients) and fell further at year 3 (three positive reports in two of six patients; data not shown). Positive autoimmunity test results were intermittent in most patients and were not associated with any clinical manifestations, except for autoimmune thrombocytopenia in patient 2.

Four patients were living in a protected environment before entering the study but were able to enter the community after gene therapy. The clinical improvements enabled patients to attend school (according to age), have normal social interactions, and to take part in sporting activities.

## Discussion

This interim analysis of data from a study of eight patients with severe Wiskott-Aldrich syndrome, six of whom have been followed up for more than 3 years, suggests that this disease can be successfully treated by one infusion of autologous CD34+ HSPCs transduced with a lentiviral vector encoding for a functional *WAS* gene, preceded by a reduced-intensity conditioning regimen. Treatment outcome was favourable for all eight patients, and all patients were alive and well at the last follow-up visit.

Preparatory conditioning was tolerated as anticipated in all eight patients. Expected chemotherapy-related severe neutropenia was transient, and all eight patients showed good haemopoietic recovery thereafter, with only one patient needing administration of granulocyte colony-stimulating factor. The infusion of autologous lentiviral vector-transduced HSPCs was well tolerated, with no adverse reactions. No immune response against the vector or WASP was detected. Sustained multilineage engraftment of genetically corrected cells was recorded in peripheral blood and bone marrow, attributable to persistent engraftment of HSPCs in the bone marrow over time. Of note, haemopoiesis is driven by distinct subsets of HSPCs, which contribute differently in early and late phases of reconstitution.^24^

Expression of vector-derived WASP in most lymphoid cells and platelets led to improvement of immune function and platelet counts. This expression resulted in clinical benefit for patients, who showed a progressive amelioration of their clinical status, with protection from severe infections and bleeding events.

It should be considered that this study has a small sample size and is based at one centre, is single arm, and is open label, using the patient's prestudy status as the control for the assessment of efficacy of gene therapy. This study design is justified by the rarity and severity of the disease and type of intervention. The study design was agreed with the EMA and its PDCO and takes EU guidelines into consideration.

With respect to safety, adverse events and serious adverse events were recorded most frequently during the first 6 months after gene therapy. These events were mainly of infectious origin. Two serious adverse events that were related to infections—both within the first 6 months after gene therapy—were life-threatening, but these events resolved with minor sequelae in one patient.^16^ Infections are not unexpected during ongoing immune reconstitution because of the time needed for engraftment, similar to patients who undergo traditional allogeneic bone marrow transplantation.^9^, ^10^ No graft failure was reported after gene therapy; graft-versus-host disease was not reported either, a finding that was expected because of the autologous nature of the procedure. No organ deterioration related to disease progression or complications or to treatment-related toxicity was seen. Importantly, malignant diseases typically reported in patients with Wiskott-Aldrich syndrome have not been detected in any of the eight patients treated to date. Intervention-free survival (ie, free from second HSPC transplant, splenectomy, or chronic immunosuppression) is 100% as of the data cutoff.

Autoimmunity is a key risk factor for a poor outcome in patients with Wiskott-Aldrich syndrome.^5^, ^18^ Despite the presence of circulating autoantibodies, clinical autoimmune manifestations reported after gene therapy were restricted to one case of transient autoimmune thrombocytopenia occurring in the early follow-up phase and one case already present before treatment. No autoimmune endocrinopathies were seen after gene therapy. Importantly, the pre-existing severe refractory autoimmune thrombocytopenia in one patient and severe autoimmune and autoinflammatory clinical manifestations in another resolved completely after treatment. Eczema was cleared in seven of eight patients; in the one patient in whom eczema did not resolve, eczema was mild and intermittent by 3-year follow-up. These results indicate that the immune dysregulation that predisposes patients with Wiskott-Aldrich syndrome to develop autoimmune manifestations could be ameliorated by insertion of the correct *WAS* gene and establishment of functional T cells and B cells. These data accord with the finding that lentiviral gene therapy corrected the alterations of both central and peripheral B-cell tolerance checkpoints and ameliorated B-cell development and functionality in patients with Wiskott-Aldrich syndrome.^25^, ^26^ Of note, the engraftment and expansion of gene-corrected B cells expressing WASP could have been favoured by pretreatment rituximab-mediated depletion of B cells, particularly autoreactive B cells, highlighting the key role of this drug in the conditioning of patients with Wiskott-Aldrich syndrome before gene therapy.

Validated quality-of-life general assessments might not adequately assess the key disease-related effects of Wiskott-Aldrich syndrome and, hence, risk underestimating the impaired quality of life of a patient with this disease. In this study, we used a simple questionnaire (appendix pp 1, 2) to assess the effect of disease burden on patients' quality of life before and after treatment, focusing in particular on life restrictions, social interactions, attendance to educational services and participation in sporting activities. Moreover, we also assessed the frequency of hospitalisation before and after gene therapy, as a measure of medical care requirement affecting patients' daily life. Overall, gene therapy resulted in a substantial improvement in quality of life for patients and their families, with patients socialising in their community with peers, attending school (according to age), starting to participate in sporting activities, and progressively reducing their time spent in hospital and their chronic medical care needs. Further work would be needed to develop quality-of-life assessments specific to Wiskott-Aldrich syndrome for implementation in a future clinical trial.

By comparison with a British and French gene therapy clinical study in patients with Wiskott-Aldrich syndrome that used a lentiviral vector structure,^27^ our results are similar with respect to improvement in immune functions and resolution of eczema and autoimmunity, but they differ with respect to the degree of HSPC engraftment, immune reconstitution, and platelet count increase. Specifically, three of four patients in the British and French trial who did not have splenectomy had platelet counts lower than 20 × 10^9^ cells per L after gene therapy (median follow-up after gene therapy, 2 years [range 9–30 months]), compared with our study in which none of the eight patients had splenectomy and all eight patients had platelet counts greater than 20 × 10^9^ cells per L after gene therapy at the last follow-up visit. Immune reconstitution was suboptimum in the British and French trial because only two of six patients with follow-up longer than 1 year stopped immunoglobulin replacement therapy and CD8+ T cells remained low in most patients. This finding contrasts with our results showing normalisation of lymphocyte counts in seven of eight patients, allowing the suspension of antimicrobial prophylaxis in all eight patients after gene therapy and discontinuation of intravenous immunoglobulin in all seven patients with follow-up longer than 1 year, followed by vaccine administration in five patients with a protective antibody response for most vaccines. Differences in disease severity, age at treatment, study conduct, and preconditioning might have contributed to the diverse outcome between the two studies. Furthermore, the specific vector manufacture and transduction processes could account for the differences seen in the correction level in vitro and in vivo. Although the CD34+ cell dose was similar (median 9·6 × 10^6^ cells per kg in our study *vs* 7·3 × 10^6^ cells per kg in the British and French trial), median vector copy number per genome in transduced CD34+ cells was 2·4 in our study and 1·3 in the British and French trial.^27^ Moreover, the degree of in-vivo gene marking in the myeloid lineage in the British and French trial was lower compared with our trial (median vector copy number per genome 0·1 in the British and French trial *vs* 0·8 in our study at the last available follow-up visit). This difference was seen despite us using a less intense dose of chemotherapy in our study.

Insertional mutagenesis and consequent leukaemia is considered a known risk related to gene therapy using integrating viral vectors. In a German study of gene therapy for Wiskott-Aldrich syndrome using a γ-retroviral vector,^11^ seven of ten treated patients developed leukaemia, which was attributed to the type of vector used in combination with a strong promoter. Leukaemias were diagnosed 1–5 years after gene therapy, with four of seven cases diagnosed within 3 years. In our study that used a lentiviral vector, no clonal expansion or leukaemia development have been recorded to date. The lack of a transcriptionally active long-terminal repeat of the self-inactivating lentiviral backbone, combined with a moderately active internal promoter to drive transgene expression, such as the WASP endogenous promoter, is a major safety advantage of the vector compared with the γ-retroviral vector used in the German Wiskott-Aldrich syndrome study.^11^ The insertion site analyses we did in our current trial show that the vector insertions are polyclonal, and there is no evidence of any aberrant selection of clones carrying insertion sites in the proximity of known oncogenes.

In the British and French clinical study,^27^ no leukaemia events were reported in patients followed up for 7–42 months. To date, more than 100 patients with various inherited diseases have been treated using lentiviral vectors and no causally related adverse events have been reported.^28^

The current accepted curative treatment for Wiskott-Aldrich syndrome is allogeneic HSPC transplantation when an HLA-identical donor is available. Historically, transplants from alternative donors were characterised by increased risk of toxicity and mortality, and larger cohorts using current alternative donor transplant protocols have not been published (eg, NCT02064933 by the Primary Immune Deficiency Treatment Consortium). Findings of two large retrospective series of patients who received HSPC transplants—an international study of 194 patients by Moratto and colleagues^9^ and a single-centre US study of 47 patients by Shin and colleagues^10^—showed overall survival of 84% and 81%, respectively, with most deaths occurring within the first 3–6 months after transplantation and severe infection being the main cause of death. In the international study,^9^ a risk factor for determining outcome was age at the time of transplant, with patients older than 5 years and an unrelated donor having a poorer outcome. In the US study,^10^ older children were most likely to die: seven of nine patients who died were older than 2 years at the time of HSPC transplantation. In patients with Wiskott-Aldrich syndrome who underwent allogeneic HSPC transplantation, complications were most common within the first year after the transplant (46%), with the main complications being infections needing hospitalisation (55 [28%] of 194 patients in the international study),^9^ autoimmune manifestations (27 [14%] of 194 patients in the international study,^9^ and 17 [55%] of 31 patients receiving a transplant after 2001 in the US study),^10^ severe (grade >2) acute graft-versus-host disease (22 [11%] of 194 patients in the international study,^9^ and between 15% and 44% of patients in the US study),^10^ and graft failure or rejection (13 [7%] of 194 patients in the international study,^9^ and three [6%] of 47 patients in the US study).^10^ Autoimmune cytopenias resolved in most patients in the international study, with median time to recovery of 14 months after HSPC transplantation.^9^ The most common cytopenia was thrombocytopenia, typically occurring within the first 6 months after HSPC transplantation in the international study.^9^ A recent improvement in outcome of HSPC transplantation in patients with Wiskott-Aldrich syndrome^29^ has been obtained owing to advances in graft manipulation techniques and optimisation of conditioning regimens.^30^

With the limitations of a single-centre study, our results are suggestive of a more benign safety profile, with fewer complications after gene therapy compared with those previously reported after HSPC transplantation.^9^, ^10^ No deaths were reported in our study, with a median age at treatment of 2·2 years (range 1·1–12·4). Gene therapy is, therefore, a potentially suitable alternative to HSPC transplantation because of the absence of graft-versus-host disease, since gene therapy is an autologous procedure and is preceded by use of a reduced-intensity conditioning regimen. This strategy opens the possibility to treat adult patients with Wiskott-Aldrich syndrome with chronic complications, for whom allogeneic transplantation would be associated with high risks; the report of successful lentiviral gene therapy in a 30-year-old patient with severe Wiskott-Aldrich syndrome manifestations supports this idea.^31^

Successful HSPC transplantation has been proven to correct thrombocytopenia in most patients. In the international study by Moratto and colleagues,^9^ HSPC transplantation resulted in a significant increase of mean platelet count at the last follow-up visit; however, in 36 (24%) of 152 patients, the platelet count did not return to normal and, in 14 (9%) patients, severe thrombocytopenia was persistent (platelet count 10–42 × 10^9^ cells per L). Analysis of variables in the study by Moratto and colleagues^9^ affecting the degree of thrombocytopenia correction after allogeneic HSPC transplantation showed a significant effect of myeloid chimerism on the reconstitution of platelet counts in patients with follow-up longer than 1 year. Although gene therapy in our study did not return platelet counts to normal, platelet levels increased significantly and platelets were of a typical size, reducing the risk of bleeding and related disorders, and no patients needed to be admitted to hospital because of bleeding events after treatment. It is noteworthy that the patients in our study had platelet counts after treatment in the range of those measured in patients with mixed donor–host chimerism after successful allogeneic bone marrow transplant (range 30 000–150 000 cells per μL).^9^ This observation suggests that, in gene therapy, a minimum level of engraftment could be needed to correct thrombocytopenia with a significant effect on reconstitution of platelet number. We noted some correlation between median platelet counts and the proportion of transduced clonogenic progenitors in bone marrow at 1 year after gene therapy (appendix p 12). Because bone marrow contains a mixed population of transduced and non-transduced HSPCs and, hence, megakaryocytes both positive and negative for WASP, and since the percentage of WASP-positive platelets in peripheral blood was considerably higher than the percentage of lentiviral vector-transduced HSPCs in bone marrow (median 76·6% [figure 4A] *vs* 42·1% [figure 2A] at 1 year of follow-up), it is possible that a selective advantage of platelets expressing WASP exists and favours their survival in the periphery or their release from bone marrow into peripheral blood.

Correction of thrombocytopenia is of paramount importance for cure of Wiskott-Aldrich syndrome, particularly for patients with milder disease in whom thrombocytopenia is the prominent feature. Therefore, efforts to improve correction of platelet counts should be made in the future. Further optimisation of the conditioning regimen could increase lentiviral vector-corrected HSPC engraftment and lead to enhanced platelet recovery. Improvement of the vector construct might be also considered but should be weighted with the potential risks of using a stronger viral promoter.^32^

Differences in stem-cell source, cell dose, purification, manipulation, and conditioning might account for the longer duration of neutropenia noted in our study compared with allogeneic transplantation.^29^, ^30^

CD34+ cells from bone marrow and mobilised peripheral blood have similar capacity to re-establish long-term haemopoiesis after allogeneic HSPC transplantation, but the use of mobilised peripheral blood is associated with a reduction in time to engraftment, probably attributable to differences in composition of progenitor cells.^33^ Studies in a larger cohort of patients are needed to establish if ex-vivo gene-corrected CD34+ cells from mobilised peripheral blood and bone marrow differ in their biological properties and capacity to restore haemopoiesis after gene therapy for Wiskott-Aldrich syndrome.

In conclusion, data from the interim analysis of our study suggest that patients with severe Wiskott-Aldrich syndrome can be treated with gene therapy to enable restoration of immune function, substantially increased platelet count, and reduction in autoimmunity; patients had strikingly reduced incidence of disease-specific clinical events without complications related to the drug product. Furthermore, these improvements were sustained or increased over the duration of follow-up, leading to substantial improvements in quality of life for patients. Longer follow-up through a dedicated registry, along with results in a larger cohort of patients with Wiskott-Aldrich syndrome, will provide further evidence of the long-term safety and efficacy of this treatment.

## Data sharing

For further information, please contact the corresponding author.
