# Supplementary material for: Lentiviral haemopoietic stem/progenitor cell gene therapy for treatment of Wiskott-Aldrich syndrome: interim results of a non-randomised, open-label, phase 1/2 clinical study
Source: Lancet Haematol. 2019 Apr 10;6(5):e239–53. doi: 10.1016/S2352-3026(19)30021-3 (PMC6494976; doi:10.1016/S2352-3026(19)30021-3)
Supplement: Supplementary appendix [file mmc1.pdf]

THE LANCET
Haematology

Supplementary appendix

This appendix formed part of the original submission and has been peer reviewed. We post it as supplied by the authors.

Supplement to: Ferrua F, Cicalese MP, Galimberti S, et al. Lentiviral haemopoietic stem/progenitor cell gene therapy for treatment of Wiskott-Aldrich syndrome: interim results of a non-randomised, open-label, phase 1/2 clinical study. *Lancet Haematol* 2019; published online April 10. http://dx.doi.org/10.1016/S2352-3026(19)30021-3.

SUPPLEMENTARY MATERIAL

METHODS

Study Design

Briefly the trial comprises (i) a screening phase during which fulfilment of inclusion/exclusion criteria were evaluated and pre-enrolment data on clinical history was were collected from patients' clinical charts; (ii) a baseline phase to assess pre-treatment clinical conditions and confirm that the patient is suitable for the treatment procedure, (iii) a treatment phase which included harvest of HSPCs from mobilised peripheral blood (PBSCs) or bone marrow (BM) or mobilised peripheral blood (PBSCs) [after GCSF ± Plerixafor]), administration of anti-CD20 monoclonal antibody and reduced intensity conditioning regimen and infusion of autologous transduced CD34+ cells; and (iv) a core follow-up period of 3 years after gene therapy with an additional 5-year follow-up until the 8th year after gene therapy. Study design is shown in Figure 1.

Outcomes

Safety

Primary safety endpoints included: the absence of engraftment failure or delayed haematological reconstitution (absolute neutrophil count [ANC] $<0{\cdot}5 \times 10^9$/L at 60 days after treatment), with aplastic bone marrow and requirement for back-up infusion; non-haematological regimen related toxicity (National Cancer Institute [NCI] CTCAE grade ≥2 for clinical manifestations; grade ≥3 for laboratory and other tests) during the first 100 days after treatment; short-term safety and tolerability of lentiviral-transduced cell infusion (serious adverse reactions within 48 hours from infusion) and long-term safety of lentiviral-transduced cell infusion (absence of replication competent lentivirus [RCL] and abnormal clonal proliferation [ACP]). Secondary safety endpoints included lack of immune response to transgene (antibodies to WASP) and overall safety surveillance of the treatment on the basis of adverse event (AE) and serious adverse event (SAE) monitoring and laboratory parameters; insertion-site (IS) analysis. (1,2)
RCL was evaluated by ELISA for HIV p24 antigen (serum), DNA PCR for VSV-G env (cells), RT-PCR for HIV-pol RNA (serum) and anti-HIV p24 antibodies.(1) ACP was monitored by clinical and laboratory surveillance, T-cell repertoire study, and bone marrow examination. Antibodies to WASP were evaluated by Western blot.

Efficacy

Primary efficacy endpoints included: overall survival; sustained engraftment of genetically corrected HSPCs in BM and peripheral blood (PB) lineages; expression of vector-derived WASP in lymphocytes and platelets; improved T-cell function; antigen-specific responses to vaccination; improved platelet count and normalization of platelet volume. Secondary efficacy endpoints included multilineage engraftment of genetically corrected cells; reduced frequency of severe infections, bleeding events and autoimmune phenomena; improvement in eczema; improvement in quality of life.
The presence of gene corrected cells was assessed by quantitative PCR (qPCR) on bone marrow and peripheral blood subpopulations.(1,3) The percentage of transduction was evaluated also on colony forming cells (CFC) originated from patient's stem cells, from which genomic DNA was extracted and VCN of each single colony evaluated, as previously described.(1) WASP expression evaluation was performed on platelets, lymphocytes, lymphocyte subsets and monocytes, by intracellular staining with an anti-human WASP antibody after cell permeabilization, as previously described.(1) In vitro T-cell proliferation upon stimulation with immobilised anti-CD3 (anti-CD3i) monoclonal antibody was measured as described previously.(1)
Severe infections and bleeding events were evaluated by clinical history for the 12-month period prior to treatment and by clinical monitoring after gene therapy (physical examination, adverse event [AE] and serious adverse event [SAE] reports, haematological and microbiological tests). Organ-specific and systemic autoantibodies (number and titre of antibody when available) and/or clinical manifestations of autoimmunity were evaluated starting from one year after gene therapy. Eczema score, as applied by Imai, K. *et al* (4), was recorded every 6 months for the first 3 years, then annually. Quality of life was assessed qualitatively by number of hospitalizations, need for protective environment, ability to attend school/kindergarten, performing sport and social life. To this aim, the following questionnaire was given to patients' parents.

Social life		
Is the patient living in a protected environment?	❑ **Yes**	❑ **No**
Is the patient attending kindergarten/school (according to age)?	❑ **Yes**	❑ **No**
How is the patient's social ability with peers?	❑ **Normal**	❑ **Abnormal**

If abnormal, pleas specify: ____

Does the patient practice any sport (if applicable for age)? ❑ **Yes** ❑ **No**

If Yes or No, comment: ____

Insertion site analyses

Genomic DNA was extracted with QIAamp DNA Blood Mini kit or Micro kit (QIAGEN) and integration sites (IS) were collected from magnetic beads-isolated BM or PB cell types through LAM-PCR and high-throughput Illumina sequencing as previously described. (1,2)
The diversity index for each sample and time point was estimated on the basis of richness (number of unique integration sites marking individual clones) and evenness (relative clonal contribution in terms of sequence reads associated to each integration site). (2)

WASP mean fluorescence intensity (MFI) in platelets

The MFI of WASP expression in platelets was calculated on total CD41a+ platelet gate. Data were acquired at Canto II flow cytometer and analysed using Flow Jo Software. The ratio between patient and control MFI was calculated. Peripheral blood from healthy controls was obtained in accordance with the 1964 Helsinki declaration and its later amendments or ethical standards. Informed consent was approved by the Institutional Ethical Committee of San Raffaele Hospital in 2009 (Tiget Periblood protocol). Twenty-three healthy controls have been recruited.

Study populations

Eczema score before gene therapy is reported in Table 1 for all patients who received this treatment (n=8). Eczema was described as mild in 5 patients, moderate in 2 or severe in 1 patient. In figure 3E, the graph shows the eczema score before GT and at 3 years of follow up in the 6 patients with follow up ≥3 years after treatment. Among them, 3 had mild eczema before gene therapy.

The term gastrointestinal bleeding includes all clinically observable bleeding events involving gastrointestinal tract, such as bloody diarrhoea and blood streaks in stools. These events were classified according to common toxicity criteria (CTC) criteria and attributed a grade from 1 (mild) to 4 (life-threatening).

Zhu clinical score, described for the first time in Zhu *et al.* (5), is a disease severity score, based on the presence of different combinations of disease characteristic clinical findings: micro-thrombocytopenia, immunodeficiency, eczema, autoimmune diseases or cancer. This simple scoring system was developed to differentiate clinical phenotypes caused by different WAS gene mutations, and was adapted over time according to the advancing knowledge of disease genotype-phenotype correlations. The table below, adapted from (6), summarized the main features corresponding to the different disease scores.

Score	XLN	iXLT	XLT		Classic WAS		
	0	<1	1	2	3	4	5
Thrombocytopenia	-	-/+	+	+	+	+	+
Small platelets	-	+	+	+	+	+	+
Eczema	-	-	-	(x)	+	++	-/(x)/+/++
Immunodeficiency	-/(x)	-	-/(x)	(x)	+	+	(x)/x
Infections	-/(x)	-	-	(x)	+	+/++	-/(x)/+/++
Autoimmunity and/or malignancy	-	-	-	-	-	-	+
Congenital neutropenia	+	-	-	-	-	-	-
Myelodisplasia	-/+	-	-	-	-	-	-

Recently, a slightly revised version of the disease score has been proposed, including the definition of a distinct WAS subgroup with the respect to Severe Refractory Thrombocytopenia, that should be considered a characteristic of severe disease with the assignment of a score 5 if present under the age of 2 years (7).

In the original protocol, the presence of a revertant cell population in T cells represented an exclusion criterion, as WASP expression in revertant T cells was not distinguishable from the expression driven by the gene therapy vector. Afterwards, in 2014 the protocol was amended to remove this exclusion criterion to allow inclusion also of patients with such characteristics, since we thought that this criterion was no longer justified. First, WASP expression from the vector had been shown in all previously treated WAS patients after gene therapy, in multiple cell lineages. Second, since older patients carry more frequently a revertant population, maintenance of this criterion would have excluded from gene therapy several patients without a compatible HSC donor and that might have benefited from this treatment. Finally, the presence of a higher background in WASP expression in T cells in patients is accounted in the analyses of WASP expression.

REFERENCES

1. Aiuti A, Biasco L, Scaramuzza S, et al. Lentiviral Hematopoietic Stem Cell Gene Therapy in Patients with Wiskott-Aldrich Syndrome. Science (80-). 2013;341(6148):1233151.
2. Biasco L, Pellin D, Scala S, et al. In Vivo Tracking of Human Hematopoiesis Reveals Patterns of Clonal Dynamics during Early and Steady-State Reconstitution Phases. Cell Stem Cell. 2016;19(1):107–19.
3. Scaramuzza S, Biasco L, Ripamonti A, et al. Preclinical safety and efficacy of human CD34(+) cells transduced with lentiviral vector for the treatment of Wiskott-Aldrich syndrome. Mol Ther. 2013 Jan;21(1):175–84.
4. Imai K, Morio T, Zhu Y, et al. Clinical course of patients with WASP gene mutations. Blood. 2004 Jan 15;103(2):456–64.
5. Zhu Q, Watanabe C, Liu T, et al. Wiskott-Aldrich Syndrome/X-linked Thrombocytopenia: WASP gene Mutations, Protein Expression, and Phenotype. Blood. 1997 Oct 1;90(7):2680–9.
6. Albert MH, Notarangelo LD, Ochs HD. Clinical spectrum, pathophysiology and treatment of the Wiskott-Aldrich syndrome. Curr Opin Hematol 2011 Jan;18(1):42–8.
7. Mahlaoui N, Pellier I, Mignot C, et al. Characteristics and outcome of early-onset, severe forms of Wiskott-Aldrich syndrome. Blood. 2013 Feb 28;121(9):1510-6.
8. Brigida I, Scaramuzza S, Lazarevic D, et al. A novel genomic inversion in Wiskott-Aldrich-associated autoinflammation. J Allergy Clin Immunol. 2016;138(2):619–622.e7.

FIGURES

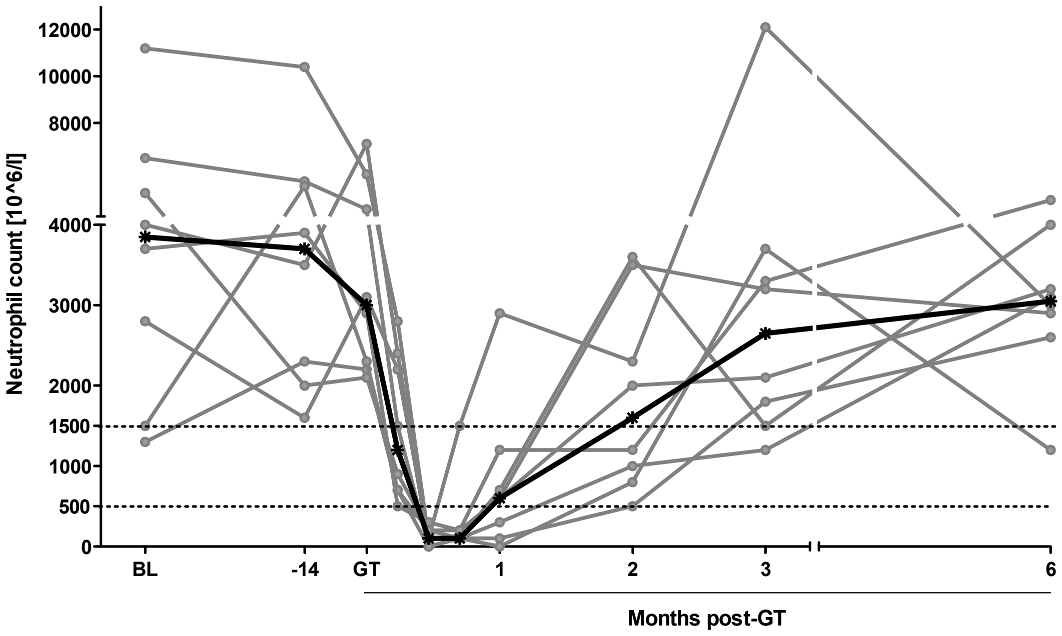

Fig. S1. Neutrophil counts in the first 6 months of follow up after gene therapy. Gene therapy was administered on day 1 (month 0). Grey lines represent individual patient data and the black line represents the median. Lower dashed line represents the limit below which neutropenia is defined severe, upper dashed line represents lower end of normal range of neutrophil count. Patient 4 experiences transient mild neutropenia at 6 month follow up, after which neutrophil count returned normal. BL, baseline; -14, -14 days before gene therapy.

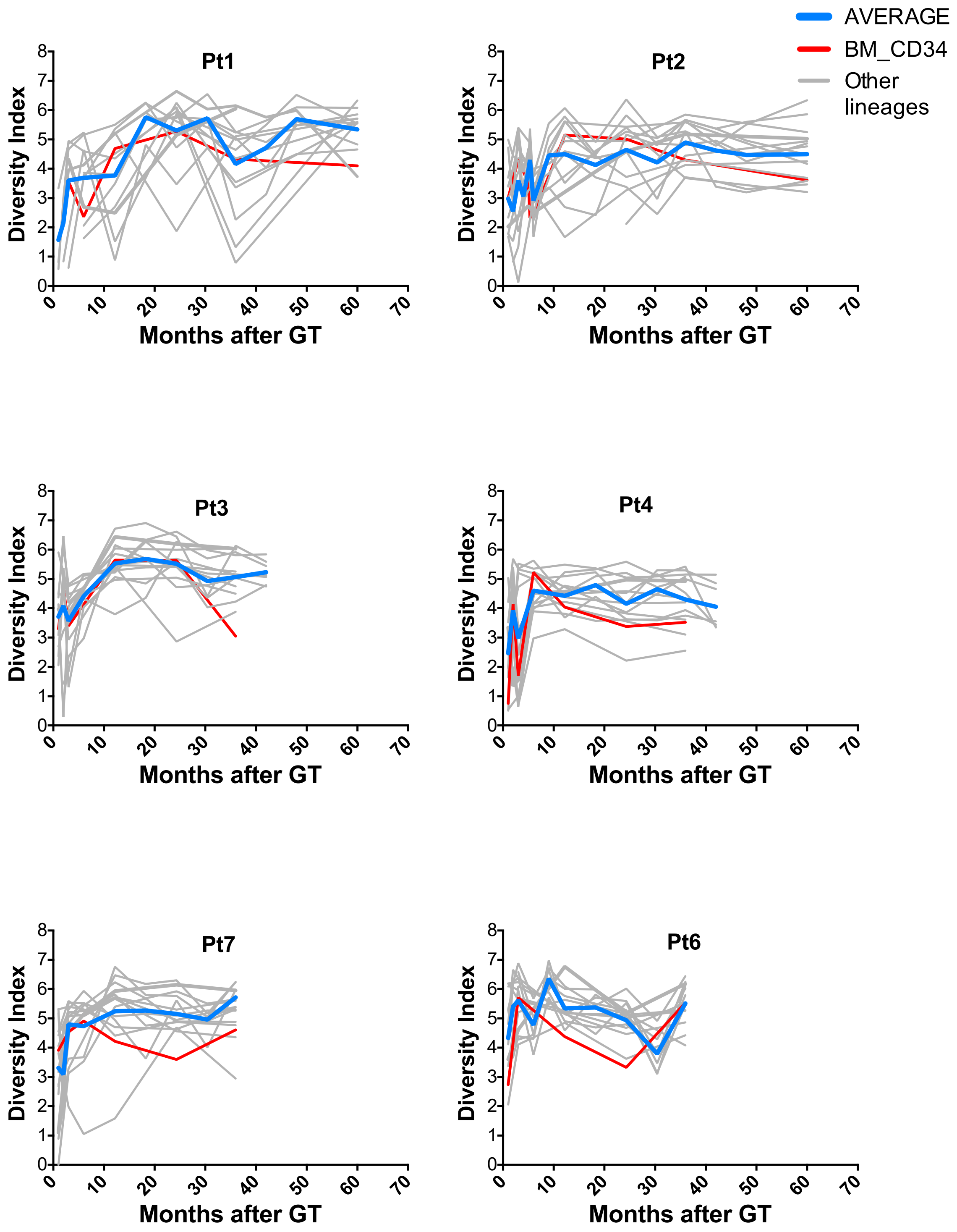

Fig S2. Diversity index calculated on the number of insertion sites and their relative sequence counts. Diversity index was calculated on data from the first six Wiskott-Aldrich syndrome patients as previously described.(2) For each plot, grey lines represent diversity of individual cell lineages, red line represents diversity of $CD34^+$ cells and blue line represents average (mean) diversity among all lineages. Pt: patient.

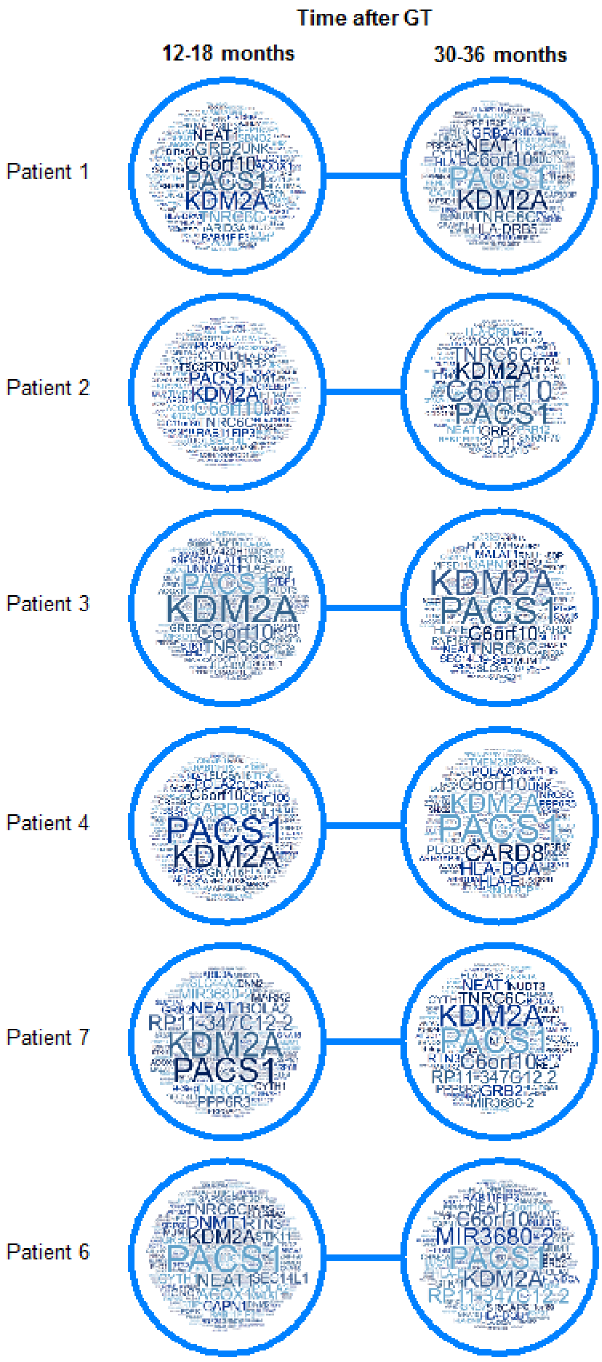

Fig S3. Insertion site analysis. Word clouds of genes proximal to the insertion sites detected within common insertion site (CIS) regions at two different time periods after gene therapy. Closest genes associated to top 5 CIS (top 5 lowest p-value according to scan statistic) are reported for the first 6 patients at early (12-18 months) and late (30-36 months) time points after gene therapy.

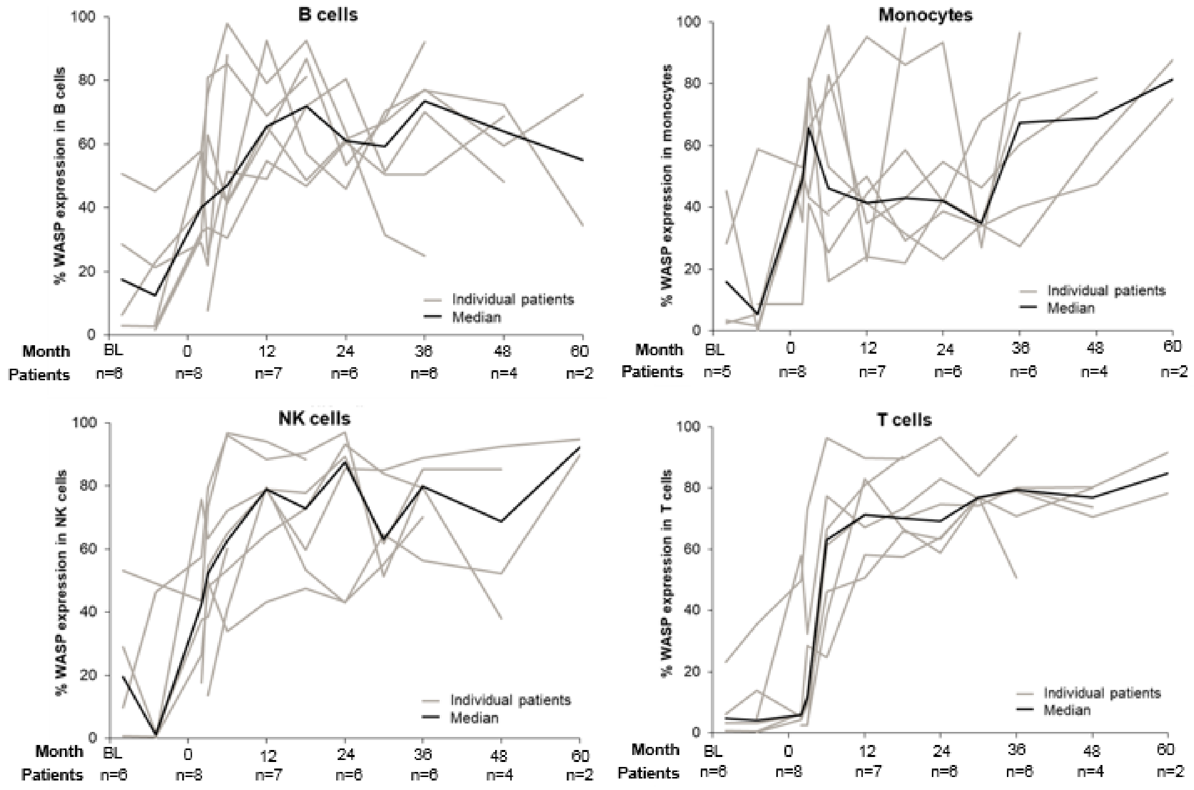

Fig. S4. WASP expression in lymphocyte subsets and monocytes measured by flow cytometry. Gene therapy was administered on day 1 (month 0). BL: baseline. For each plot, grey lines represent individual patient data and the black line represents the median.

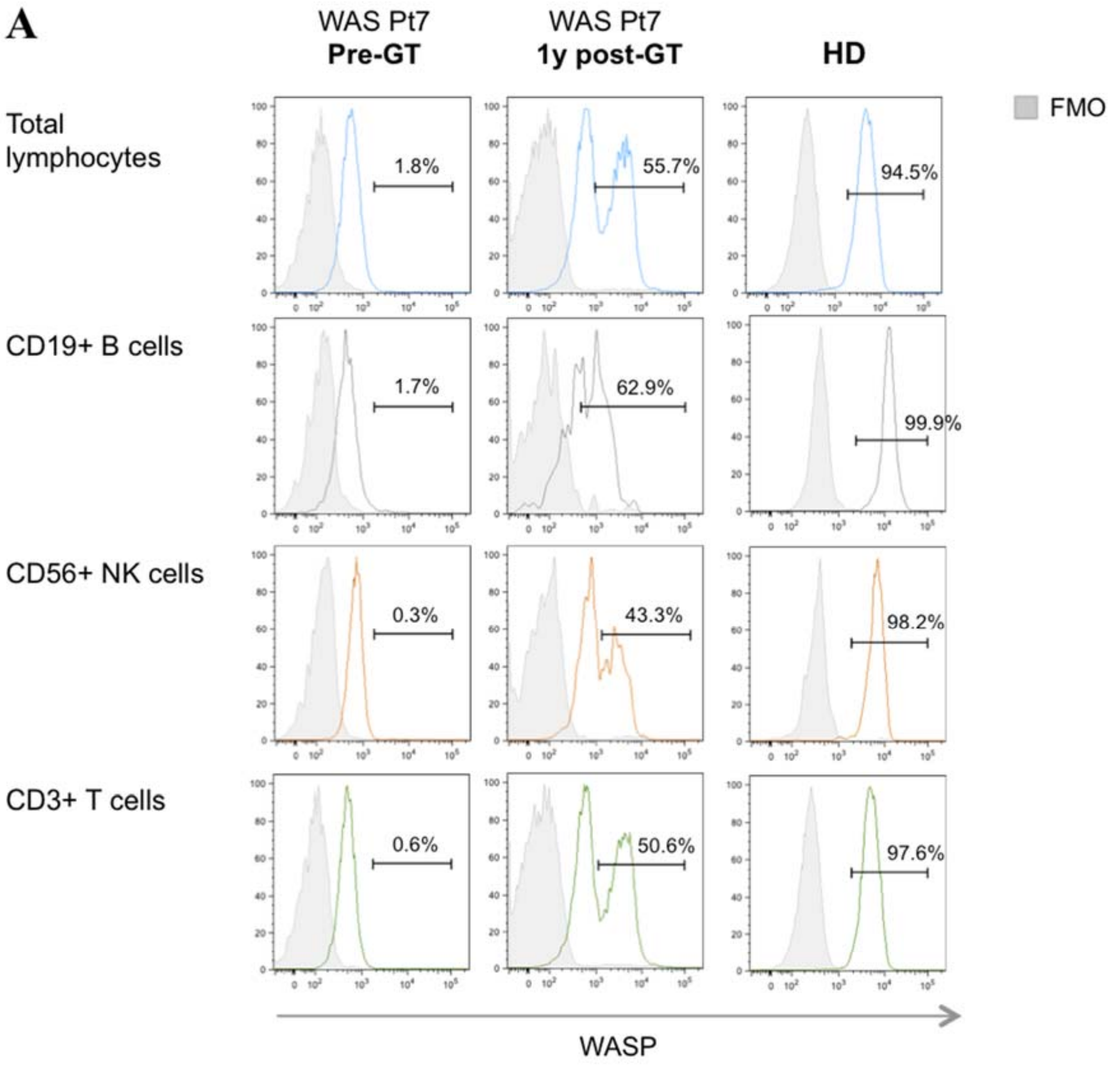

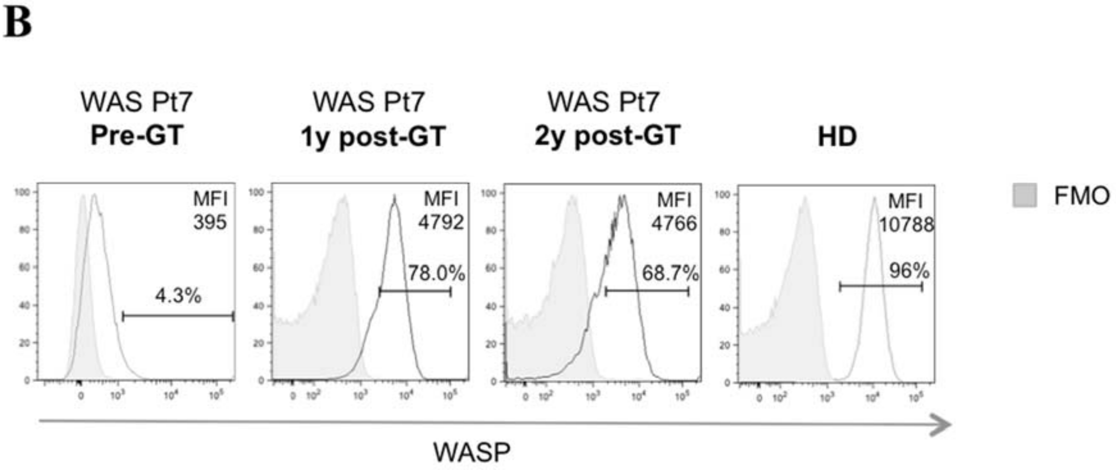

Fig. S5. WASP expression in lymphocyte subsets and platelets before and after GT. Representative flow cytometric analyses of WASP expression in Patient (Pt) 7 before and after GT on lymphocyte subsets (A) and platelets (B). Shaded plot: fluorescence minus one (FMO) control staining; solid coloured plot: WASP staining on the indicated subpopulations. Numbers indicate the percentage (%) of positive cells. HD, healthy donor; y, year.

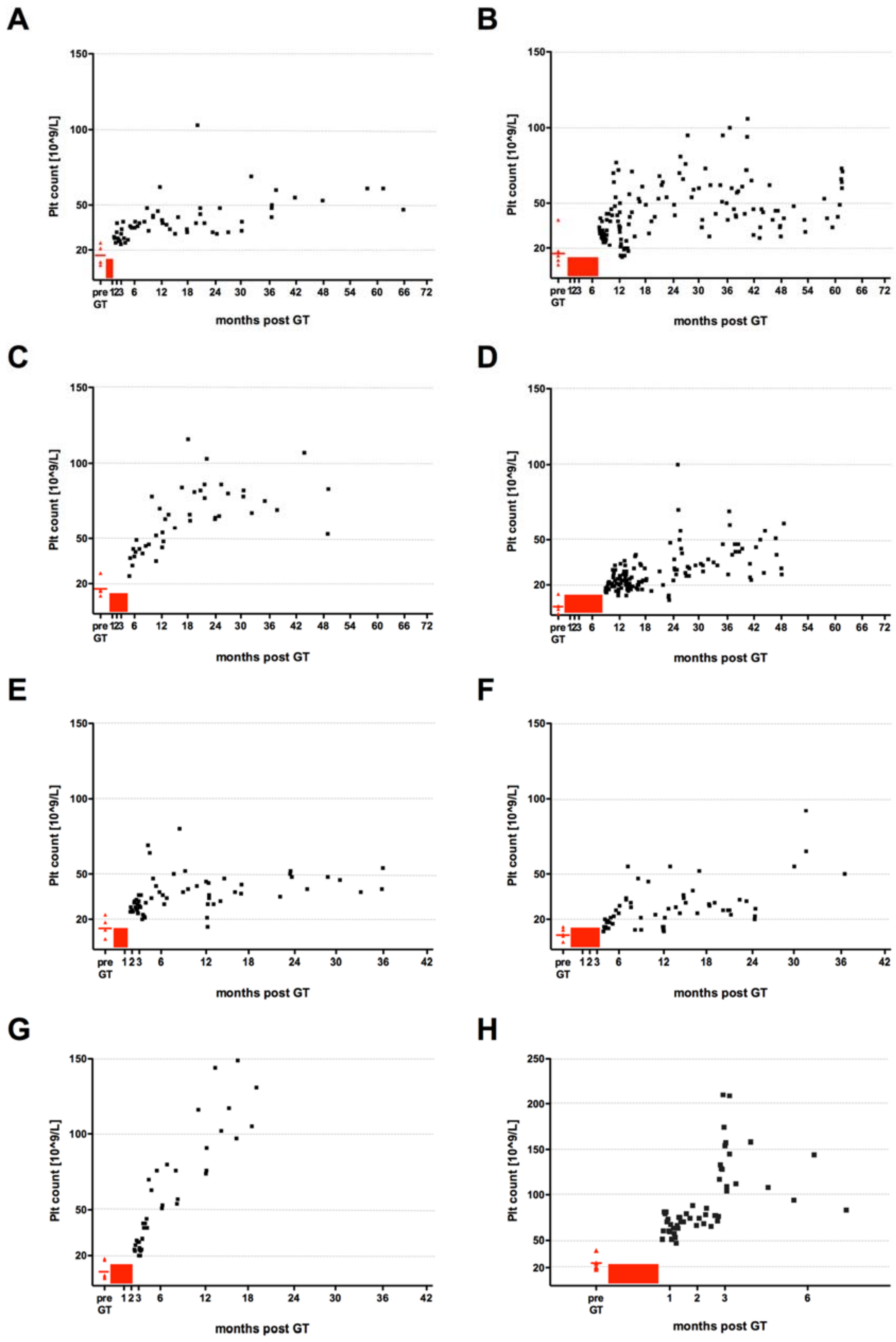
A
B
C
D
E
F
G
H
Plt count [10^9/L]
months post GT
pre GT

Fig S6. Individual plots of platelet count by time. Platelet count data for follow up periods available for the interim analysis are shown for patients 1, 2, 3, 4, 7, 6, 8 and 9 in panel A, B, C, D, E, F, G and H, respectively (the x- and y-axis scale is not the same for all patients and depends on the duration of follow up and maximum platelet count, respectively). Red bar represents the length of transfusion dependence after treatment. During this interval, platelet counts were censored because they were not representative of patients' own platelet production. Only platelet counts >7 days after platelet transfusions were included in the analysis after treatment. As regards the period before gene therapy, platelet counts in the 3 months before starting the treatment phase were collected with the largest feasible time interval from platelet transfusions, whose frequency was determined by clinical need.

A

B

C

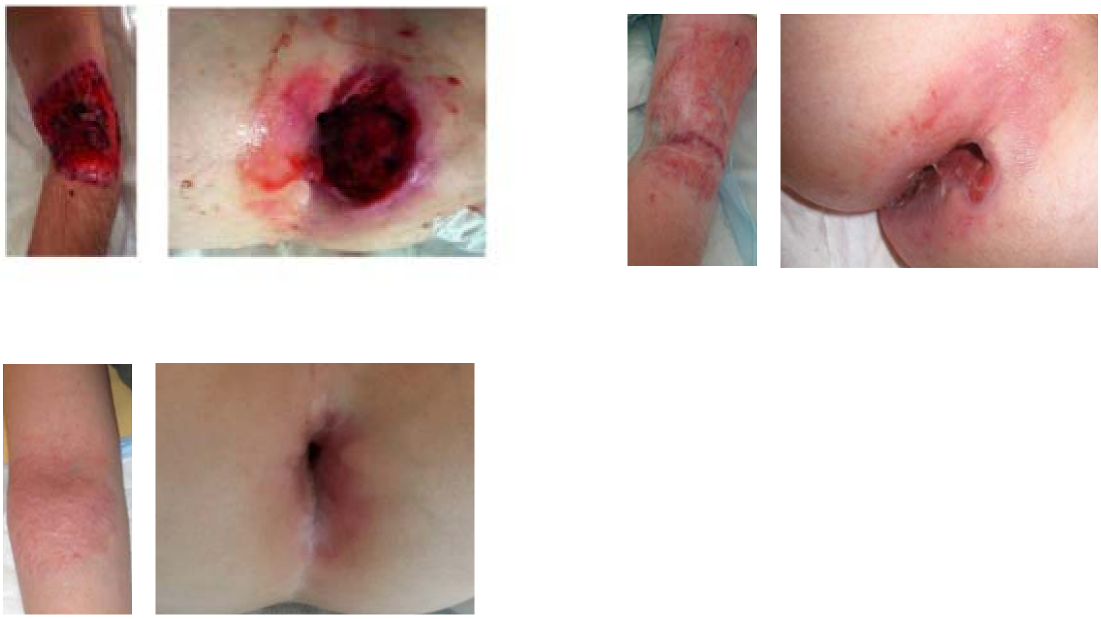

Fig S7. Evolution of severe pyoderma gangrenosum in patient 9. Panel A shows the patient at the time of Wiskott-Aldrich syndrome diagnosis [modified from *Brigida et al., JACI 2016* (8)]. Panel B shows improvement of pyoderma gangrenosum after 2 months of treatment with Anakinra. Panel C shows persistent resolution of pyoderma gangrenosum 7 months after gene therapy, with the patient off immunosuppressive treatment.

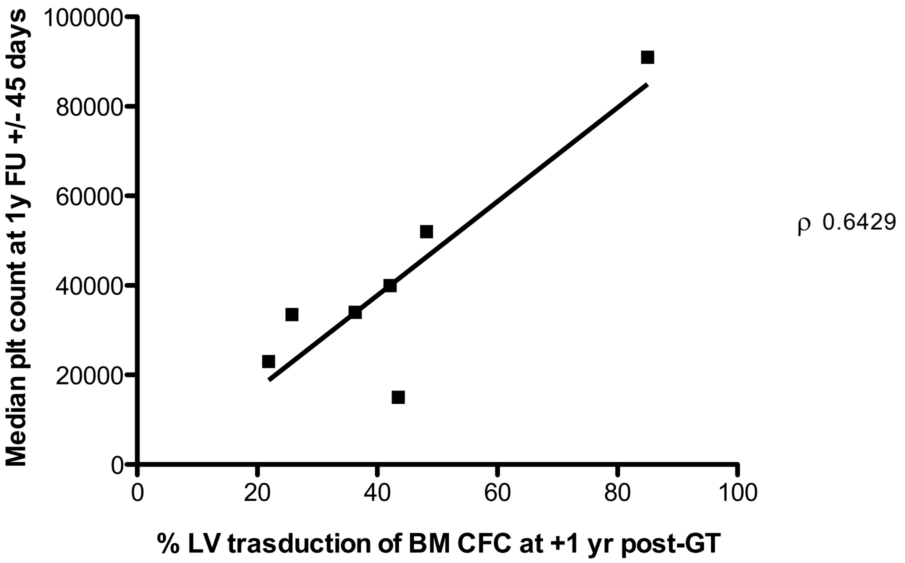

Fig. S8. Correlation between median platelet (plt) count at 1 year (yr) follow up (FU) after gene therapy (GT) and LV-gene corrected cell engraftment in BM progenitors. LV, lentivirus; BM, bone marrow; CFC, colony forming unit. ρ, Spearman's rank correlation coefficient.

TABLES

Table S1 Myeloablation: neutropenia and busulfan chemotherapy

Patient	Nadir neutropenia: Day post-GT	Nadir neutropenia: ANC (10^9/L)	Engraftment onset (day post-GT)*	Days with ANC <0.5 x 10^9/L	Days with ANC <0.1 x 10^9/L	Busulfan: AUC after first dose (ng x h/mL)	Busulfan: AUC after fifth dose (ng x h/mL)	Busulfan: Estimated cumulative AUC (ng x h/mL)	Busulfan: Total dose (mg/kg)	G-CSF
1	17	0·00	25	12	2	5310	6208	46072	7·6	No
2	12	0·20	25	17	0	3544	NA	33314	9·4	No
3	14	0·00	31	19	7	3571	7174	42980	10·1	No
4	16	0·00	45	32	23	3938	4337	36000†	12·3	Yes††
6	14	0·00	59	51	20	5221	5891**	44448	9·6	No
7	14	0·00	29	17	6	7764	6134**	55592	7·4	No
8	17	0·07	42	29	4	4744	6668**	45648	9·6	No
9	14	0·08	18	5	1	4988	6135**	44492	7·7	No

ANC: absolute neutrophil count; AUC: area under the curve; GT: gene therapy; NA: not available
*First of 3 consecutive days with ANC ≥0·5 x10^9/L. **After sixth dose. †After 9 doses of busulfan. ††G-CSF administered on days 42 and 44 for delayed neutrophil recovery thought to be caused by intense response to the conditioning regimen and concomitant infection.
Notes: Patients 1, 2 and 3 have been described previously.[14] The target cumulative busulfan AUC was 36,000-48,000 ng x h/mL for the first 6 treated patients. Following a protocol amendment, the target cumulative busulfan AUC was changed to 48,000 (±10%) ng x h/mL for the final 2 treated patients. Cumulative AUC was estimated as (AUC1+AUC6)x4 for patients who received 8 doses of busulfan and (AUC1+AUC6)x4+D9xAUC6/D5 for patients who received 9 doses of busulfan.

Table S2 Total follow up post gene therapy for each patient at time of data cut-off date

Patient	Follow up (years)
1	5·6
2	5·3
3	4·1
4	4·4
6	3·0
7	3·0
8	1·5
9	0·5

Table S3 **Total lymphocyte and lymphocyte subset absolute counts before and after gene therapy**

	Total lymphocyte absolute count [*10^9/L]							
	**Patient 1**	**Patient 2**	**Patient 3**	**Patient 4**	**Patient 6**	**Patient 7**	**Patient 8**	**Patient 9**
**Screening**	**3·52**	10·68	**4·53**	2·55	1·57	**2·73**	**1·35**	**1·08**
**Baseline**	**1·65**	**5·10**	**3·25**	**2·05**	1·01	2·05	0·57	0·65
**Day -14**	**1·51**	**3·59**	**3·02**	0·89	0·86	0·62	0·89	0·83
**Day +1**	0·28	1·69	0·76	0·18	0·27	0·25	0·27	0·21
**Day +14**	0·66	0·96	0·49	0·27	0·12	0·24	0·32	0·45
**Day +30**	**1·14**	1·86	2·05	0·43	0·22	0·64	0·43	**1·50**
**Day +60**	**1·14**	2·41	**2·80**	0·60	0·49	1·56	0·42	0·80
**Day +90**	**1·91**	2·39	0·32	0·77	0·79	1·65	0·54	**1·50**
**Day +180**	**2·03**	**2·47**	**4·88**	0·89	1·47	1·33	**1·13**	**1·50**
**Year +1**	**2·60**	**3·37**	**4·33**	1·14	1·57	**2·39**	**1·12**	
**Year +1.5**	**2·80**	**2·85**	7·55	**1·86**	**1·85**	1·38	**1·63**	
**Year +2**	**2·42**	**3·47**	**4·33**	1·65	**2·00**	**2·09**		
**Year +2.5**	**3·25**	**3·34**	8·82	**2·07**	**2·11**	**2·05**		
**Year +3**	**3·30**	**3·16**	**5·16**	**1·43**	**2·02**	1·31		
**Year +4**	**2·78**	**2·89**	**4·47**	**2·71**				
**Year +5**	**2·82**							

	B cell absolute count [*10^9/L]							
	**Patient 1**	**Patient 2**	**Patient 3**	**Patient 4**	**Patient 6**	**Patient 7**	**Patient 8**	**Patient 9**
**Screening**	0·18	**1·19**	**0·74**	**0·31**	0·39	**0·85**	**0·22**	0·03
**Baseline**	0·11	**0·62**	**0·81**	**0·33**	0·24	**0·71**	0·09	0·02
**Day -14**	0·00	0·01	0·01	0·00	0·00	0·00	0·00	0·00
**Day +1**	0·00	0·00	0·00	0·00	0·00	0·00	0·00	0·00
**Day +14**	0·00	0·02	0·02	0·00	0·00	0·00	0·00	0·00
**Day +30**	0·00	0·06	0·09	0·00	0·00	0·03	0·00	0·00
**Day +60**	0·04	0·17	**0·74**	0·03	**0·25**	**0·64**	0·00	0·09
**Day +90**	0·05	0·38	0·11	0·17	**0·35**	**0·78**	0·12	**0·35**
**Day +180**	0·15	0·13	**1·42**	0·04	**0·34**	**0·52**	**0·27**	**0·46**
**Year +1**	**0·34**	**0·38**	**1·56**	0·15	**0·37**	**0·84**	0·19	
**Year +1.5**	**0·24**	**0·35**	2·49	**0·27**	**0·39**	**0·44**	**0·34**	
**Year +2**	**0·23**	**0·31**	**0·91**	**0·24**	**0·42**	**0·58**		
**Year +2.5**	**0·28**	**0·26**	**1·90**	**0·21**	**0·35**	**0·55**		
**Year +3**	**0·33**	**0·30**	**0·74**	0·18	**0·32**	**0·35**		
**Year +4**	**0·32**	**0·20**	**0·71**	**0·33**				
**Year +5**	**0·31**	**0·25**						

	NK cell absolute count [*10^9/L]							
	**Patient 1**	**Patient 2**	**Patient 3**	**Patient 4**	**Patient 6**	**Patient 7**	**Patient 8**	**Patient 9**
**Screening**	**0·22**	1·64	**0·43**	**0·48**	0·19	**0·39**	**0·21**	**0·42**
**Baseline**	**0·17**	**0·80**	0·19	**0·27**	**0·14**	**0·28**	0·07	0·23
**Day -14**	**0·11**	nd	**0·23**	**0·12**	**0·22**	**0·14**	**0·13**	**0·24**
**Day +1**	nd	0·07	0·02	0·01	0·01	0·00	0·01	0·01
**Day +14**	0·01	0·04	0·01	0,03	0·00	0·01	0·01	0·00
**Day +30**	0·04	**0·11**	0·04	0·03	0·04	0·10	0·03	**0·10**
**Day +60**	**0·11**	**0·20**	0·10	**0·10**	0·05	**0·28**	**0·10**	**0·12**
**Day +90**	**0·21**	**0·22**	0·01	0·07	**0·13**	**0·25**	**0·08**	**0·30**
**Day +180**	**0·14**	**0·17**	**0·13**	**0·19**	**0·17**	**0·17**	**0·20**	**0·09**
**Year +1**	0·08	**0·17**	0·05	**0·15**	**0·11**	**0·33**	**0·11**	
**Year +1.5**	0·08	**0·16**	**0·19**	**0·28**	**0·11**	**0·11**	**0·27**	
**Year +2**	0·08	**0·23**	**0·10**	**0·33**	0·08	**0·12**		
**Year +2.5**	**0·25**	**0·28**	**0·49**	**0·49**	**0·10**	**0·20**		
**Year +3**	**0·17**	**0·27**	**0·21**	**0·45**	**0·13**	**0·10**		
**Year +4**	**0·15**	**0·23**	**0·14**	**0·52**				
**Year +5**	**0·08**	**0·31**						

	T cell absolute count [*10^9/L]							
	**Patient 1**	**Patient 2**	**Patient 3**	**Patient 4**	**Patient 6**	**Patient 7**	**Patient 8**	**Patient 9**
**Screening**	**3·05**	**7·95**	**2·90**	**1·45**	0·87	1·29	**0·84**	0·47
**Baseline**	**1·31**	**3·83**	**1·99**	1·33	0·60	1·02	0·35	0·35
**Day -14**	**1·34**	**3·23**	**2·64**	0·70	0·54	0·44	0·50	0·41
**Day +1**	0·27	**1·62**	0·70	0·16	0·25	0·23	0·24	0·15
**Day +14**	0·64	0·88	0·46	0·23	0·12	0·22	0·31	0·44
**Day +30**	**1·06**	**1·68**	**1·84**	0·38	0·15	0·46	0·39	1·34
**Day +60**	**0·91**	**1·95**	**1·77**	0·37	0·15	0·55	0·30	0·53
**Day +90**	**1·48**	**1·72**	0·18	0·45	0·21	0·58	0·29	0·78
**Day +180**	**1·65**	**2·09**	**2·81**	0·57	**0·92**	0·56	0·52	**0·93**
**Year +1**	**2·13**	**2·63**	**2·60**	0·78	**1·13**	**1·22**	0·61	
**Year +1.5**	**2·45**	**2·23**	4·91	**1·26**	**1·34**	0·88	**0·87**	
**Year +2**	**2·06**	**2·75**	**3·42**	**1·02**	**1·51**	**1·33**		
**Year +2.5**	**2·72**	**2·59**	6·36	**1·24**	**1·58**	1·17		
**Year +3**	**2·81**	**2·46**	**3·96**	**0·79**	**1·50**	0·86		
**Year +4**	**2·30**	**2·36**	**3·51**	**1·60**				
**Year +5**	**2·40**	**2·56**						

	T helper cell (CD3+CD4+) absolute count [*10^9/L]							
	**Patient 1**	**Patient 2**	**Patient 3**	**Patient 4**	**Patient 6**	**Patient 7**	**Patient 8**	**Patient 9**
**Screening**	**1·96**	**2·07**	**1·50**	**1·02**	0·76	**1·07**	**0·64**	0·25
**Baseline**	**0·74**	**1·40**	**1·23**	**1·02**	0·50	0·84	0·27	0·19
**Day -14**	**0·86**	**1·37**	**1·58**	**0·55**	0·45	0·34	0·39	0·26
**Day +1**	0·23	0·82	0·61	0·14	0·22	0·20	0·20	0·13
**Day +14**	**0·49**	0·37	0·25	0·18	0·10	0·16	0·25	0·20
**Day +30**	**0·62**	0·40	0·51	0·16	0·12	0·29	0·32	0·33
**Day +60**	**0·47**	0·54	0·67	0·22	0·13	0·39	0·23	0·23
**Day +90**	**0·70**	0·54	0·10	0·34	0·17	0·46	0·22	0·27
**Day +180**	**0·89**	**0·66**	0·84	0·22	**0·59**	0·41	0·31	**0·59**
**Year +1**	**1·07**	**0·96**	**1·38**	0·41	**0·73**	**0·81**	0·37	
**Year +1.5**	**1·32**	**0·89**	**2·33**	**0·77**	**0·81**	**0·60**	**0·51**	
**Year +2**	**0·89**	**1·17**	**1·75**	**0·59**	**0·96**	**0·90**		
**Year +2.5**	**1·16**	**1·20**	2·57	**0·71**	**0·81**	**0·74**		
**Year +3**	**1·33**	**1·01**	**1·73**	**0·47**	**0·86**	**0·54**		
**Year +4**	**1·00**	**0·95**	**1·36**	**0·77**				
**Year +5**	**1·30**	**1·07**						

	Cytotoxic T cell (CD3+CD8+) absolute count [*10^9/L]							
	**Patient 1**	**Patient 2**	**Patient 3**	**Patient 4**	**Patient 6**	**Patient 7**	**Patient 8**	**Patient 9**
**Screening**	**0·92**	4·70	**1·29**	**0·43**	0·06	0·19	0·16	0·20
**Baseline**	**0·55**	**1·98**	**0·67**	0·28	0·05	0·14	0·06	0·15
**Day -14**	**0·43**	**1·47**	**0·98**	0·15	0·04	0·08	0·10	0·13
**Day +1**	0·03	**0·65**	0·09	0·02	0·02	0·02	0·03	0·02
**Day +14**	0·11	**0·40**	0·19	0·05	0·02	0·06	0·04	**0·23**
**Day +30**	**0·35**	**1·08**	**1·30**	0·22	0·02	0·15	0·06	**1·01**
**Day +60**	**0·38**	**1·22**	**1·06**	0·15	0·02	0·13	0·05	**0·29**
**Day +90**	**0·68**	**1·00**	0·07	0·11	0·03	0·10	0·06	**0·38**
**Day +180**	**0·67**	**1·24**	**1·59**	**0·34**	0·16	0·14	0·13	**0·29**
**Year +1**	**0·91**	**1·52**	**1·00**	**0·35**	0·22	**0·36**	0·14	
**Year +1.5**	**0·97**	**1·23**	2·09	**0·46**	0·28	0·25	**0·27**	
**Year +2**	**1·04**	**1·39**	**1·38**	**0·40**	**0·31**	**0·36**		
**Year +2.5**	**1·26**	**1·27**	3·31	**0·49**	**0·48**	**0·32**		
**Year +3**	**1·30**	**1·32**	1·97	0·29	**0·32**	0·24		
**Year +4**	**1·07**	**1·36**	1·86	**0·72**				
**Year +5**	**0·91**	**1·42**						

	CD4+ Naïve T cell (CD4+CD45RA+) absolute count [*10^9/L]							
	**Patient 1**	**Patient 2**	**Patient 3**	**Patient 4**	**Patient 6**	**Patient 7**	**Patient 8**	**Patient 9**
**Screening**	**1·28**	**1·07**	**0·97**	0·65	0·53	0·80	**0·34**	nd
**Baseline**	nd	0·57	0·85	**0·57**	0·39	0·80	0·14	0·08
**Day +30**	**0·43**	0·18	0·33	0·09	0·06	0·22	0·20	0·12
**Day +60**	0·27	0·23	0·30	0·14	0·07	0·30	0·13	0·08
**Day +90**	**0·35**	0·21	0·06	0·26	0·06	0·30	0·11	0·04
**Day +180**	**0·41**	0·09	0·27	0·10	0·31	0·24	0·10	**0·30**
**Year +1**	**0·54**	0·40	**1·10**	0·23	**0·47**	**0·68**	0·17	
**Year +1.5**	**0·73**	0·39	2·05	**0·46**	**0·55**	0·39	0·19	
**Year +2**	**0·53**	**0·57**	**1·28**	0·37	**0·65**	**0·61**		
**Year +2.5**	**0·72**	**0·43**	1·81	0·37	**0·51**	0·39		
**Year +3**	**0·77**	**0·48**	1·11	0·20	**0·53**	0·27		
**Year +4**	**0·44**	**0·38**	**0·44**	0·31				
**Year +5**	0·29	**0·36**						

nd, not done.

Table S4 **Serum immunoglobulin level before and after gene therapy**

	**Pt1**		**Pt2**		**Pt3**		**Pt4**		**Pt6**		**Pt7**		**Pt8**		**Pt9**	
**Serum level**	*Pre*	*Post*	*Pre*	*Post*	*Pre*	*Post*	*Pre*	*Post*	*Pre*	*Post*	*Pre*	*Post*	*Pre*	*Post*	*Pre*	*Post*
IgG [g/L]	5·21	5·77	16·66[§]	*na*	14·01[§]	**7·00**	**9·86**[§]	4·88	**5·41**[§]	*na*	**7·80**[§]	4·11	**11·01**[§]	**7·56**	5·00[§]	*na*
IgA [g/L]	**1·51**	**1·18**	2·13	*na*	4·18	4·49	**1·25**	**1·07**	**0·69**	*na*	**1·63**	**0·9**	3·38	**2·12**	5·91	*na*
IgM [g/L]	0·11	0·33	**2·23**	*na*	**0·59**	**1·12**	**0·57**	**0·77**	0·16	*na*	0·19	0·31	0·21	**0·72**	0·29	*na*

Serum immunoglobulin level before gene therapy (at screening) and after gene therapy (at last available follow up), at least 3 months after stopping immunoglobulin replacement therapy) is reported. In bold are reported normal values for age (according to Burgio GR, Perinotto G, Ugazio AG 1991). IgM level improved in all patients after gene therapy, although it did not reach normal level for age in all. *na*, not available (patients still on immunoglobulin replacement therapy or <3 months since stopping replacement therapy).
[§] Ongoing immunoglobulin replacement therapy.

Table S5 – Antibody response to vaccination

	Protective antibody level (Y/N)				
**Vaccination antigen [U.M.]**	**Pt1**	**Pt3**	**Pt4**	**Pt7**	**Pt8**
Tetanus-IgG (ELISA) [IU/ml]	**Y** [0·45]	**Y** [1·59]	**Y** [3·07]	**Y** [5·94]	**Y** [3·63]*
Diphterie-IgG (ELISA) [IU/ml]	**Y** [1·55]	**Y** [1·95]	**Y** [2·29]	**Y** [2·2]	**Y** [1·96]*
Bordetella Pertussis-IgG [U.A.]	**N** [9·4]	**Y** [24·7]	**Y** [19·6]	**Y** [15·1]	**Y** [26·1]*
Haemophilus-IgG [ug/ml]	**Y** [3·12]	**Y** [1·34]	**Y** [>9]	**Y** [9]	**Y** [>9]*
HBs-IgG [mU/ml]	**Y** [125]	**Y** [1661]	**Y** [8858]	**Y** [8214]	**Y** [1625]*
Pneumococcus IgG *(response to Pneumovax®/Prevenar®)*	**N** [1:027]/ **Y** [1:211]	**N** [1:20]/ **Y** [1:316]*	**N** [1:165]*/ **Y** [1:434]*	**N** [1:128]°/ **N** [1:138]*	**Y** [1:250]*/ nd
Pneumococcus IgM *(response to Pneumovax®/Prevenar®)*	**N** [1:090]/ **Y** [1:420]	**N** [1:36]/ **Y** [1:516]*	**Y** [1:524]*/ **Y**[1:1819]*	**Y** [1:109]°/ **Y** [1:115]*	**Y** [1:420]*/ nd

In the table are reported specific antibody levels after 3 doses of hexavalent vaccination, 2 doses of Pneumovax® or 2-3 doses of Prevenar® vaccine, received by patients >3 months after ceasing IVIG supplementation. Specific antibody response after vaccination was performed at Immunologische Tagesklinik in Wien (Austria). Pt2, Pt6 and Pt9 were vaccinated after data cut off. After data cut off, the 5 patients reported in the table received measles, mumps and rubella (MMR) vaccine and 2 of them also varicella (VZV) vaccination, showing protective antibody titers when tested. *After data cut off. °After one dose of Pneumovax® and one dose of Prevenar®. nd - Not yet evaluable data about antibody response to Prevenar® (*ongoing*).

Vaccination antigen	Normal level
Tetanus-IgG (ELISA)	>=0·4 IU/ml
Diphteriae-IgG (ELISA)	>=0·4 IU/ml
Bordetella Pertussis-IgG	>=11 U.A.
Haemophilus-IgG	>=1 µg/ml
HBs-IgG	>= 100 mU/ml
Pneumococcus IgG	Serum titer >= 1:200
Pneumococcus IgM	Serum titer >= 1:100
